# Optic disc and cup segmentation using Vision Mamba UNet with enhanced edge-guided attention in fundus images

**DOI:** 10.1186/s12886-026-05371-x

**Published:** 2026-10-23

**Authors:** Lili Cai, Keke Hou, Zhong Mo

**Affiliations:** School of Medicine and Health, Guangzhou Xinhua University, Guangzhou, 510520 China

**Keywords:** Glaucoma, Optic disc and cup segmentation, Vision Mamba, Edge-guided attention, Fundus images

## Abstract

**Background:**

Glaucoma is an irreversible blinding disease for which early detection is essential. Precise segmentation of the optic disc (OD) and optic cup (OC) enables accurate cup-to-disc ratio (CDR) calculation, which serves as an important reference for glaucoma evaluation. However, the ambiguous boundaries between these structures make manual segmentation time-consuming and highly subjective.

**Methods:**

This study proposes a hybrid framework for OD and OC segmentation in fundus images. The framework first adopts YOLOv10 to achieve coarse localization and extraction of the OD region, followed by fine-grained segmentation using the proposed EEGA-VMUNet network. Based on a Vision Mamba-UNet backbone, we design an enhanced edge-guided attention (EEGA) module to adaptively integrate multi-scale contextual information. This module strengthens the representation of edges and fine structural features. A multi-scale loss function is also used to improve robustness to scale variations.

**Results:**

Experiments on four benchmark datasets demonstrate that EEGA-VMUNet achieves competitive segmentation performance. On the same-domain test sets, OD Dice coefficients range from 95.20% to 97.65% and OC Dice coefficients range from 87.51% to 89.95%. Zero-shot cross-domain experiments achieve an OD Dice of 95.18% and an OC Dice of 85.13%.

**Conclusion:**

The proposed model achieves competitive performance in both OD and OC segmentation tasks. Bland-Altman analysis suggests that the derived vertical cup-to-disc ratio (VCDR) holds potential as a diagnostic aid for glaucoma screening, although its precision may require further improvement for longitudinal monitoring.

**Supplementary Information:**

The online version contains supplementary material available at https://doi.org/10.1186/s12886-026-05371-x.

## Introduction

The global burden of ocular diseases has been increasing steadily. A 2024 WHO statistics report shows that around 2.2 billion people globally live with some form of vision loss. Nearly half of them come from preventable causes [1]. Among these ocular disorders, glaucoma stands as a primary contributor to irreversible blindness. According to the analysis by Tham et al. [2], glaucoma affects more than 76 million people globally. By 2040, this figure is projected to exceed 110 million. The disease starts silently and gets worse over time. Patients often have no symptoms until the damage is irreversible [3]. In clinical practice, the CDR is a frequently adopted metric for large-scale glaucoma screening [4]. When the number is higher than 0.5, it often points to a greater chance of having glaucoma [5]. The precision of CDR measurement depends largely on the segmentation quality of the OD and OC. Generally, the OD is simpler to delineate as it contrasts strongly with the adjacent retinal tissue. In contrast, OC segmentation is more difficult. Its boundaries are unclear due to low contrast within the optic disc. A further challenge arises from domain shift [6], a phenomenon primarily caused by variances in imaging devices and acquisition protocols. This variability degrades the generalization capability and precision of segmentation models. Classic segmentation methods include threshold-based approaches [7], edge detection techniques [8], and morphological operations [9]. These methods are simple to implement, but their performance is highly sensitive to image quality and depends heavily on hand-crafted features.

Deep learning has made a big difference in this area over the past few years. Many researchers now turn to convolutional neural network (CNN) to pull out image features on their own. Sevastopolsky [10] made U-Net lighter for OD and OC segmentation by reducing the filter count across every convolutional layer. The network still gave good results despite being smaller. Changing the model structure is not the only way. Researchers also add attention mechanisms in the network. These mechanisms let the network focus on the most important parts of the input image [11]. Guo et al. [12] proposed FAU-Net, which employs dual attention to facilitate more effective feature extraction and fusion. Self-attention is a key part of transformer models. It lets the model directly connect any two positions in the input. This helps capture long-range relationships and brings together global context [13]. Fang et al. [14] proposed UT-Net, which combines U-Net with a Transformer. The model also includes a multi-head attention mechanism to better capture context information. Peng et al. [15] combined the Swin Transformer with UNet++. This hybrid architecture keeps the benefits of CNN and adds self-attention mechanism. So when it comes to segmenting the OD and OC, the results are more accurate. However, Transformer-based models have inherent limitations. Self-attention is good for long-range work, but it demands heavy computation, and this demand goes up sharply with image size [16]. That makes it less suitable for dense prediction tasks like segmentation. In addition, several studies have explored the use of generative adversarial network (GAN) for OD and OC segmentation [17]. GANs rely on adversarial learning. But in theory, we cannot fully guarantee that they will converge or find a stable equilibrium. To train a GAN successfully, we need to keep a careful balance between the two networks. But keeping them that way is hard, the training often ends up unstable [18].

To improve OD and OC segmentation accuracy, the model needs to understand context information well. The methods mentioned above each have their own strengths for segmentation. However, they also have limitations, such as high computational complexity and difficulty in training. The introduction of state space models (SSMs) [19] offers a promising way to get around that limitation. SSMs like Mamba can model long-range dependencies in sequences or spatial data efficiently. One key strength is that their computing speed adapts to the input’s complexity, which makes data handling faster [20]. Since then, many new versions of the Mamba model have come out. For instance, the U-Mamba [21] integrates a designed CNN-SSM block into convolutional layers. This helps it segment 3D abdominal organs with good precision. VMamba [22] takes the good parts from both CNN and Vision Transformer (ViT). It works with linear complexity and still holds onto a global receptive field. The Vision Mamba UNet (VM-UNet) effectively integrates the U-Net [23] and VMamba baseline models. Both the encoding and decoding stages of the U-Net are equipped with visual state space (VSS) mechanism. This helps model long-range context efficiently while also cutting down on computation [24]. Motivated by this design, we propose EEGA-VMUNet, which integrates the enhanced edge-guided attention (EEGA) module to address the specific challenges of OD and OC segmentation. Our key contributions can be summed up below: (a) We build upon the standard EGA framework by integrating GAM and DAM with a refined module configuration, which we refer to as the EEGA module. This design is tailored to the specific requirements of OD and OC segmentation in fundus images. We embed this configuration into the VMUNet encoder-decoder framework and systematically evaluate the contribution of each component through ablation studies. (b) The EEGA module is placed in the skip connections of the encoder-decoder network to enhance informative features in both spatial and channel dimensions. This allows the model to focus on task-relevant regions and suppress irrelevant background information. The ablation results demonstrate that this design contributes to learning more reliable boundaries of the OD and OC. (c) For high-resolution images, we adopt YOLOv10 as a preprocessing step to detect and crop the region of interest (ROI). This reduces computational cost and minimizes background interference for the subsequent segmentation network. (d) We design a multi-scale loss function that combines Dice loss and cross-entropy loss to jointly supervise region segmentation and edge detection during training. (e) We evaluate our method on four public fundus datasets and under cross-dataset settings. The results show that our method achieves performance comparable to several state-of-the-art segmentation methods across most evaluation metrics.

## Methods

### Overview

**Fig. 1:**
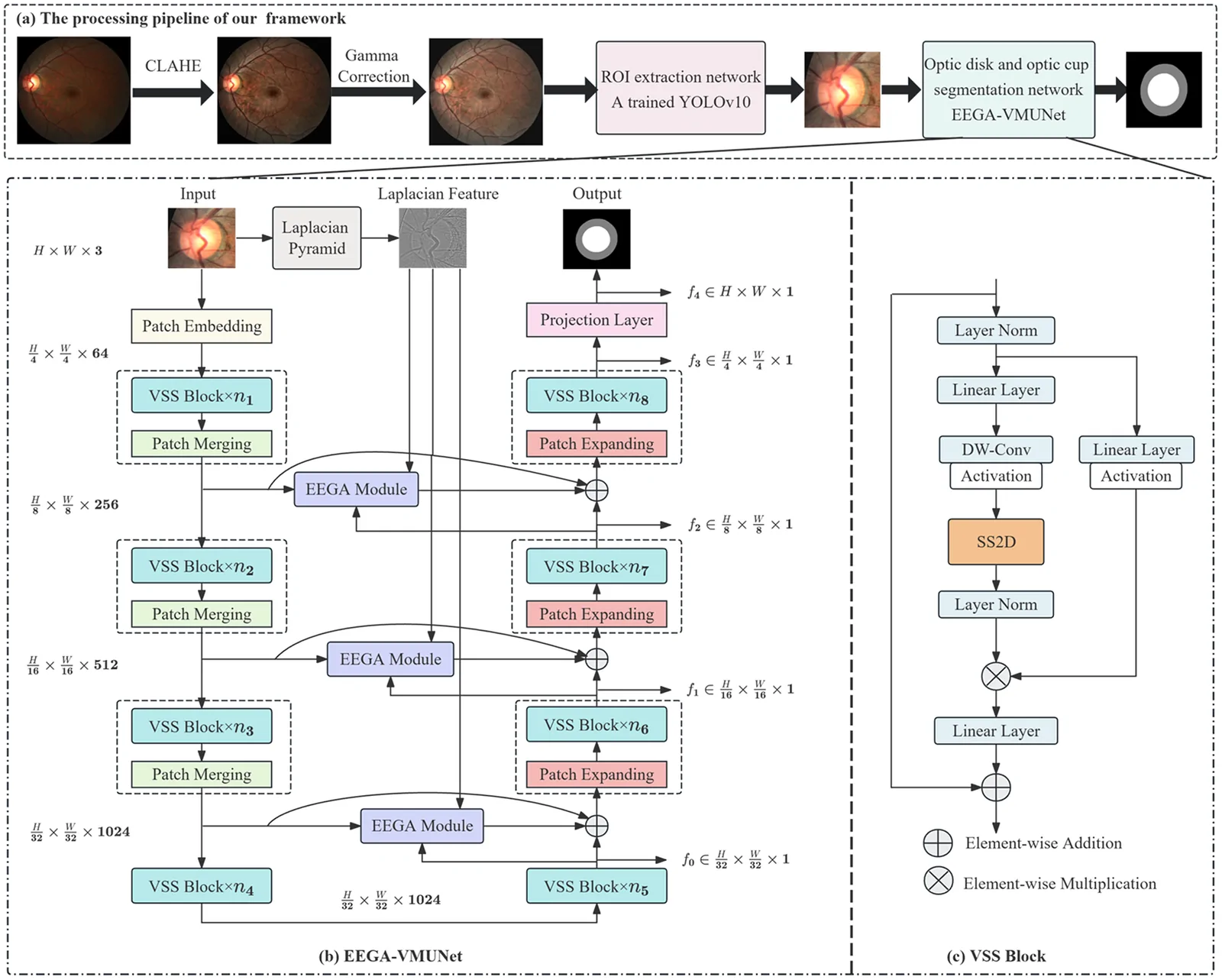
The overview of our framework. (**a**) The processing pipeline of our framework, (**b**) The structure of our EEGA-VMUNet, and (**c**) VSS block

This section details the proposed EEGA-VMUNet segmentation framework, illustrated in Fig. 1. Figure 1(a) shows the overall processing pipeline, Fig. 1(b) presents the architecture of EEGA-VMUNet, and Fig. 1 expands the internal structure of the VSS block. The architecture consists of two main parts: an ROI preprocessing stage and a segmentation network. We use a pre-trained YOLOv10 model [25] to extract the ROI from high-resolution fundus images. Then the proposed segmentation network processes these cropped ROI images. The network has a patch embedding layer, an encoder, skip connections, an EEGA module, a decoder, and a final projection layer.

### Preprocessing and ROI extraction

**Fig. 2:**
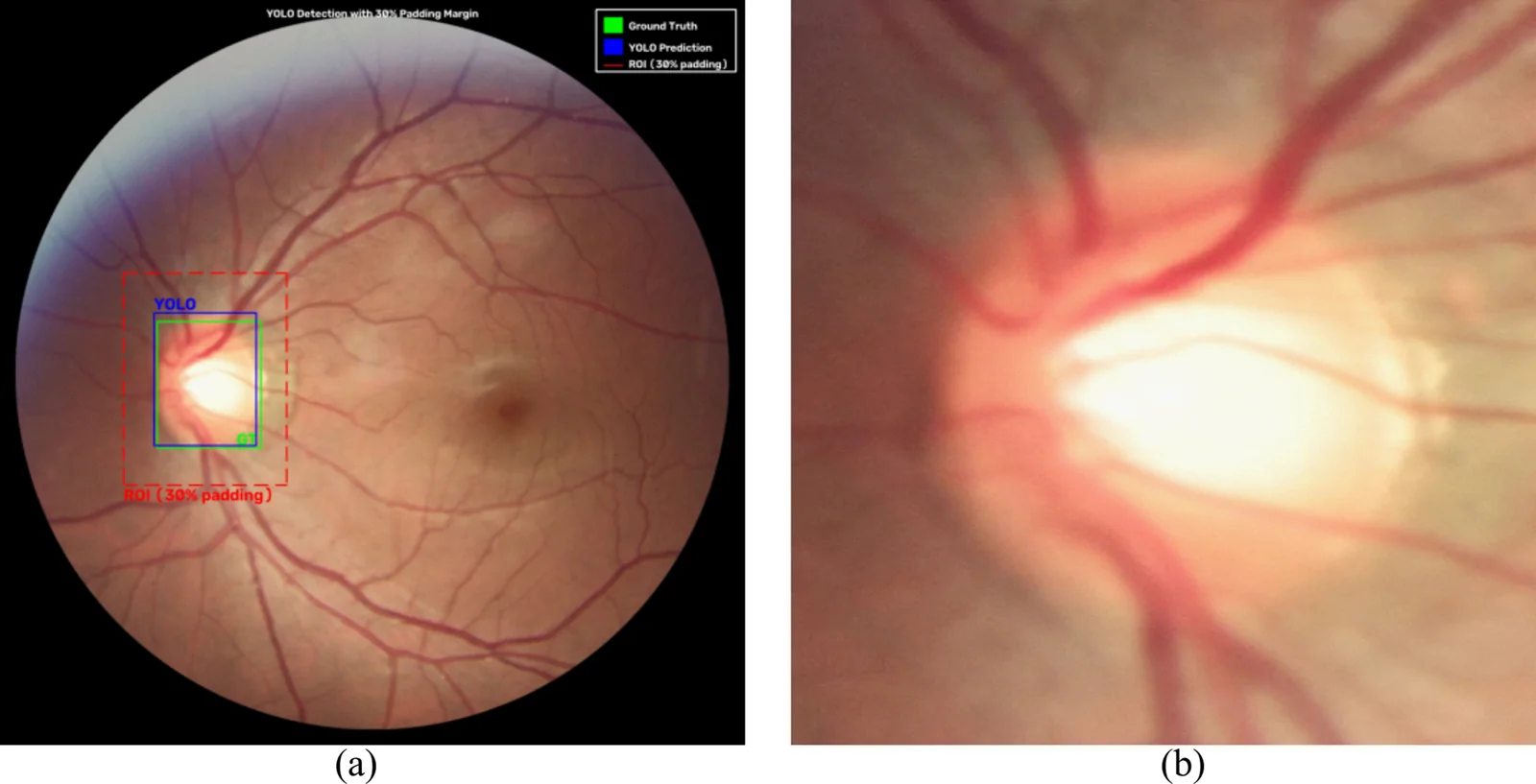
Coarse localization of the OD using YOLOv10 on the REFUGE dataset. (**a**) YOLO detection with 30% padding, (**b**) ROI patch

The color fundus images we used are typically high-resolution and show large variation in size. Most of the image consists of background, with only a small part taken up by the OD and OC. Directly inputting the full-resolution images into a segmentation network adversely affects both computational efficiency and accuracy. Therefore, localizing the ROI area is a key preprocessing step before segmentation. Among the four public datasets, the REFUGE dataset provides full-field high-resolution images that require coarse localization. For the other three datasets, we used pre-cropped versions where the optic disc is already roughly centered. Thus, the following description of ROI extraction applies specifically to the REFUGE dataset. To improve input clarity and consistency, a series of preprocessing operations is necessary. Contrast limited adaptive histogram equalization (CLAHE) [26] is an image processing algorithm designed to enhance local contrast. It operates by limiting the amplification of noise while preserving critical image details. As illustrated in Fig. 1, the original fundus images first undergo CLAHE processing, then we run gamma correction to improve the contrast where the image is underexposed. This preprocessing pipeline enhances overall image contrast and the clarity of fine details. Then the ROIs are localized and extracted from the preprocessed images. This step performs coarse localization, a task we assigned to a YOLOv10 model. The model was trained on the REFUGE training set comprising 400 full-field high-resolution fundus images. It serves as a coarse localization and ROI extraction tool rather than a precise detector, with its sole purpose being to generate a bounding box around the OD for subsequent cropping. The YOLO architecture was first introduced by Redmon et al. [27]. It has gained significant attention for its efficiency and performance in object detection. Improvements in speed and accuracy made later YOLO versions prevalent in industrial applications. For this task, we chose the YOLOv10 to ensure high accuracy without sacrificing computational efficiency. Since the OC is anatomically contained within the OD, extracting the ROI primarily involves localizing the OD region. We annotated bounding boxes around the optic disc regions using the LabelMe tool and saved the annotations in YOLO format for model training. On the REFUGE validation set (400 images), the model achieved an mAP@0.5 of 0.9944, a precision of 0.9924, and a recall of 0.9788, demonstrating high localization accuracy. To mitigate the impact of minor localization offsets, we apply a 30% padding margin to the detected bounding box before cropping. We further verified that with this padding strategy, the cropped ROI completely contained the optic disc in 100% of the REFUGE images, confirming that the coarse localization errors are effectively absorbed. Then, every extracted ROI patch is resized to 256 × 256 pixels to ensure consistent segmentation. Figure 2 shows an example of this padding strategy, where the ground-truth disc (green), the YOLO detection (blue), and the padded ROI (red dashed) are visualized for comparison. It can be observed that the padded ROI consistently encloses the entire optic disc even when the YOLO detection exhibits slight offsets, confirming the effectiveness of our padding strategy.

### EEGA-VMUNet architecture

The Mamba model is a novel deep learning architecture belonging to the class of SSM. It excels at handling long-range dependencies and complex sequential data. Mamba does well on tasks like language understanding and time series analysis. It uses a selective scan approach and this makes the computing cost grow linearly with sequence length, so it cuts down complexity without losing much accuracy. Building on this foundation, VM-UNet was developed and has demonstrated superior performance in segmenting skin lesion images [24]. Benefiting from this architecture, we illustrate the overall design of EEGA-VMUNet in Fig. 1. In our network, we adopt VM-UNet as the backbone. For optic disc and cup segmentation, this property is beneficial in several aspects. The optic cup boundary is often partially obscured by overlapping blood vessels or degraded by low contrast. In such cases, global structural information, such as the overall shape and size of the disc, can provide useful contextual cues when local intensity features are insufficient. In addition, the anatomical relationship between the disc and cup is defined not only by local pixel values but also by their relative spatial positions. Modeling long-range dependencies allows the network to integrate such global context during prediction. As shown in Fig. 1, the model includes a patch embedding layer, an encoder branch on the left, a decoder branch on the right, an EEGA module, skip connections, and a final projection layer.

The VSS block is based on the discretization theory of SSMs and serves as the core unit in both the encoder and decoder. It employs linear ordinary differential equations (ODEs) with a timescale parameter to convert continuous systems into discrete sequences. The core component of VSS is the 2D Selective Scan (SS2D) module, which scans images along horizontal, vertical, and diagonal directions to capture global context with linear time complexity. The VSS block contains two parallel branches. The main branch uses a 3 × 3 depthwise convolution for local features and the SS2D module for global context. The auxiliary branch preserves nonlinear responses through a SiLU activation and the final output is obtained by element-wise multiplication of these two branches, thus integrating global and local details.

According to Fig. 1, The patch embedding layer starts by splitting the input image into a sequence of non-overlapping patches. The encoder comprises four modules to extract hierarchical features. The first three modules share the same design. Each one contains two VSS blocks, and then a patch merging layer comes after them. This layer downsamples the feature maps in stages. The fourth module also has two VSS blocks, but it skips the patch merging step. This helps improve the feature representations further. The decoder also contains four modules and the number of VSS blocks in the bottom-up pathway is configured as [2, 2, 2, 1], respectively. Only the bottom VSS block’s input skips the patch expanding operation. The other three layers each include a patch expanding layer to gradually upsample the features and restore spatial resolution. To better use the multi-scale features, each block’s output goes through a convolutional layer. This produces a decoding map at the corresponding resolution. Given that the encoder’s feature extraction process inevitably results in partial information loss, this is especially problematic for segmenting the intricate boundaries of the OD and OC. Therefore, we introduce an EEGA module to preserve fine-grained details. As illustrated in the Fig. 1, the EEGA module is situated between corresponding hierarchical levels of the encoder and decoder. Its inputs consist of the encoded features from the encoder pathway, the decoded features from the decoder pathway at the same level, and an edge feature map obtained through edge detection. The output of the EEGA module is fused with feature maps from both the encoder and decoder at corresponding scales, and subsequently processed by VSS blocks to perform upsampling. This operation enhances conventional skip connections by preserving critical input information. Consequently, it facilitates high-quality reconstruction in the decoder stage. At the end, a projection layer takes the reconstructed features and turns them into a prediction map. This output has the same width and height as the original input.

### EEGA module

**Fig. 3:**
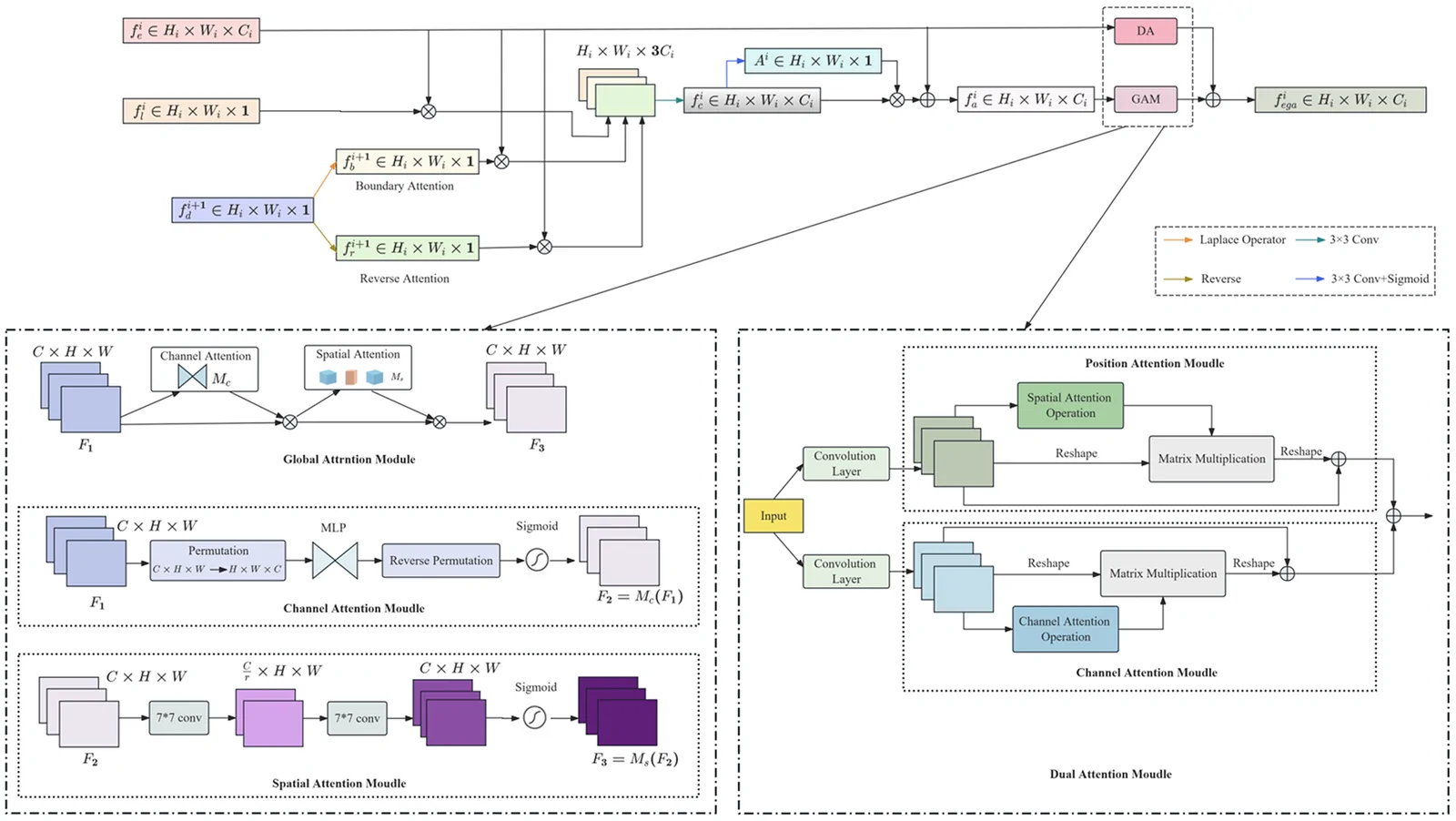
The structure of the proposed EEGA module

The structure of the proposed EEGA module is presented in Fig. 3. Based on the original EGA mechanism [28], we explored a hybrid attention strategy by combining global and detail branches for the challenges of fundus image analysis. The modifications include replacing the CBAM in the latter part of the structure with a global attention module (GAM), and incorporating a dual attention module (DAM) for enhanced feature extraction and fusion. This design helps preserve critical channel and spatial information while effectively suppressing vascular noise. Additionally, it captures the boundaries for the OD and OC from the input features to guarantee the accuracy of the final results.

Structurally, the EEGA module is positioned between corresponding levels of the encoder and decoder. This allows it to fuse outputs and facilitate high-quality feature reconstruction via skip connections. The EEGA module takes three inputs: encoded features from the encoder at the i*th* layer $f_e^i\in R{^{{H_i} \times {W_i} \times {C_i}}}$, edge features obtained from the original input using Laplacian pyramid decomposition $f_l^i\in R{^{{H_i} \times {W_i} \times 1}}$, and decoded feature maps from the previous-scale decoder layer (i + 1)*th*, denoted as $f_d^{i + 1}\in R{^{{H_i} \times {W_i} \times 1}}$. To better illustrate the generation of the edge feature $f_l^i$, we provide the algorithmic pseudocode in Algorithm 1. The boundary attention map $f_b^{i + 1}\in R{^{{H_i} \times {W_i} \times 1}}$ is derived by applying the Laplace operator to $f_d^{i + 1}$, i.e., $f_b^{i + 1} = Laplace\left( {f_d^{i + 1}} \right)$. The reverse attention map $f_r^{i + 1}\in R{^{{H_i} \times {W_i} \times 1}}$ is computed as $f_r^{i + 1} = 1 - f_d^{i + 1}$, which helps refine higher-level predictive feature maps. Subsequently, three feature maps of identical size are generated by taking the element-wise product of $f_e^i$ with $f_l^i$, $f_b^{i + 1}$, and $f_r^{i + 1}$. Here, $f_l^i$, $f_b^{i + 1}$, and $f_r^{i + 1}$ are single-channel spatial attention maps that are broadcast along the channel dimension. Specifically, each single-channel map is replicated ${C_i}$ times to match the channel dimension of $f_e^i$ before element-wise multiplication. Then we concatenate and process them through a convolutional layer to produce $f_c^i\in R{^{{H_i} \times {W_i} \times {C_i}}}$. The following equation describes this process:

1 $$f_c^i = Conv\left( {Concat\left( {f_e^i{\rm{}} \otimes f_l^i{\rm{}},f_e^i \otimes f_b^{i + 1},f_e^i \otimes f_r^{i + 1}} \right)} \right)$$

Segmentation boundaries can include extraneous information and noise. Therefore, adding the attention masking mechanism helps the model prioritize salient regions and suppress this interference. The GAM’s input $f_a^i\in R{^{{H_i} \times {W_i} \times {C_i}}}$ is calculated as the formula:

2 $$f_a^i = f_e^i + f_c^i \otimes {A^i},where{A^i} = Sigmoid\left( {Conv\left( {f_c^i} \right)} \right)$$

Here, ${A^i}\in R{^{{H_i} \times {W_i} \times 1}}$is broadcast along the channel dimension to match the channel number of $f_c^i$ before element-wise multiplication. Finally, we derive two parallel branches that employ the GAM and DAM. Specifically, the encoder feature $f_e^i$ is fed into the DAM branch to obtain $f_{da}^i = DAM(f_e^i)$, while the aggregated feature $f_a^i$is fed into the GAM branch to obtain $f_{gam}^i = GAM\left( {f_a^i} \right)$. Then we add the two branch outputs, $f_{da}^i\in R{^{{H_i} \times {W_i} \times {C_i}}}$ and $f_{gam}^i\in R{^{{H_i} \times {W_i} \times {C_i}}}$, together element by element. This gives us the final optimized decoder features $f_{ega}^i\in R{^{{H_i} \times {W_i} \times {C_i}}} = f_{da}^i + f_{gam}^i$. Both GAM and DAM effectively integrate channel and spatial attention. Since relying on a single attention type can limit feature learning, our design combines these two hybrid mechanisms to better capture feature correlations between boundary and background regions.

**Figure:**
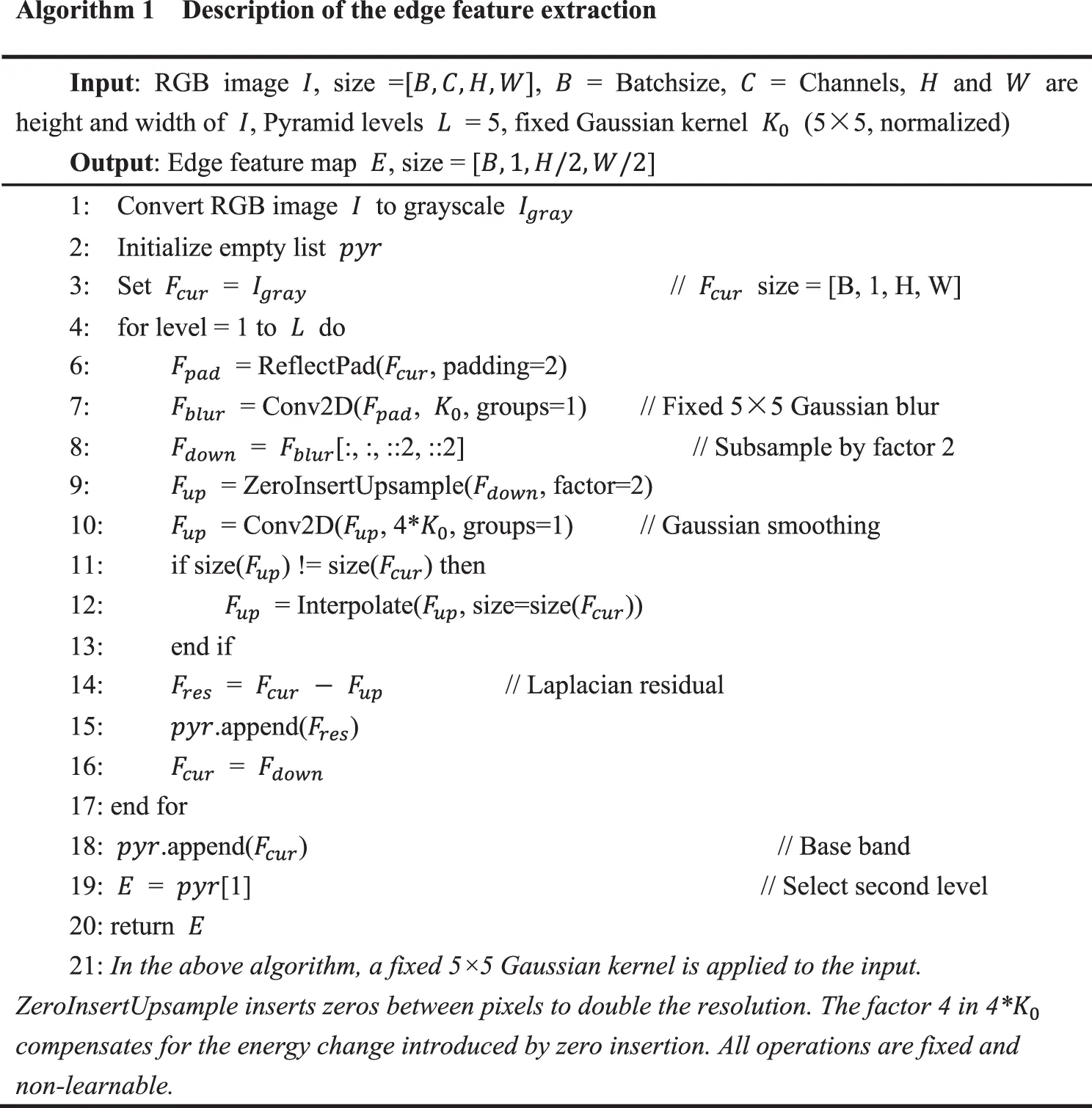

### Global attention mechanism and dual attention

Attention mechanisms have been widely explored to enhance computer vision tasks. Liu et al. [29] proposed a novel global attention mechanism, termed GAM. GAM preserves channel and spatial information by reducing information diffusion and strengthening global interactive features. As shown in Fig. 3, GAM draws inspiration from the classical convolutional block attention module (CBAM). It uses a channel attention mechanism followed by a spatial attention one. The input feature map is first refined by the channel attention sub-module. This refined map is then element-wise multiplied with the original input. Subsequently, the result is processed by the spatial attention sub-module, followed by another element-wise multiplication to produce the final output. Although structurally similar to CBAM, GAM differs in its specific implementation. Specifically, GAM first reshapes the input feature map via a 3D permutation, followed by a two-layer MLP to redistribute channel-wise information. Conversely, the spatial attention module (SAM) employs two convolutional layers to capture spatial details. This complementary design enables precise feature adjustment.

The dual attention mechanism was first applied in the dual attention network (DANet) [30]. It captures semantic interdependencies across both spatial and channel dimensions. Spatially, features at all positions are aggregated through a weighted sum to highlight informative regions. In the channel dimension, features across channel maps are integrated to emphasize interdependent channel relationships. The outputs of these two modules are then summed to refine the feature representation. Given its modular and lightweight design, the dual attention module can be directly integrated into existing frameworks [31].

### Objective function

The proposed loss function is a weighted sum of individual losses computed separately for the OC and OD. To mitigate the trade-off between pixel-wise similarity and regional consistency, we use a combined loss function computed as the mean of the Cross-Entropy and Dice losses. They are defined as follows:

3 $${L_1} = - {1 \over N}\mathop \sum \limits_{i = 1}^N \left( {{t_i}\log {p_i} + \left( {1 - {t_i}} \right) \times \log (1 - {p_i}} \right))$$

4 $${L_2} = 1 - {{2\mathop \sum \nolimits_{i = 1}^N {t_i}{p_i}} \over {\mathop \sum \nolimits_{i = 1}^N {t_i} + \mathop \sum \nolimits_{i = 1}^N {p_i}}}$$

5 $$Loss = {1 \over 2}\left( {Los{s_1} + Los{s_2}} \right)$$

Where ${p_i}$ denotes the model’s estimate of how likely a pixel is to be inside the target region; ${t_i}$ represents the true label of the pixel, and *N* is the total number of pixels. To get the total loss, we first get the loss for OC and the loss for OD separately. Then we add them up, each multiplied by a weight. The formula is given below:

6 $$Loss = {w_1}{L_{OD}} + {w_2}{L_{OC}}$$

Where ${L_{OD}}$ and ${L_{OC}}$ are the losses for the OD and OC. ${w_1}$ and ${w_2}$ are their corresponding weighting coefficients. As segmenting both structures is equally critical, we set ${w_1}$ = ${w_2}$ = 0.5. Furthermore, as depicted in Fig. 1, our network generates feature maps at multiple scale levels. To leverage this multi-scale information fully, we downsample the ground truth labels to make each output feature map have the same spatial size. A scale-specific loss is computed at each level, and these individual losses are summed to form the final total objective loss.

7 $$\begin{aligned} Los{s_{Total}} = Loss\left( {{t_0},{f_0}} \right) + Loss\left( {{t_1},{f_1}} \right) + Loss\left( {{t_2},{f_2}} \right) \\ + Loss\left( {{t_3},{f_3}} \right) + Loss\left( {t,{f_4}} \right) \end{aligned}$$

Here, ${t_0}$, ${t_1}$, ${t_2}$, and ${t_3}$ are derived by downsampling the original ground truth label *t* by factors of 32, 16, 8, and 4, respectively. By computing losses across different feature scales, the multi-scale loss function avoids the drawbacks of a single-scale approach. This helps retain fine-grained details and reduces sensitivity to scale, thus boosting segmentation results. To justify the equal weighting strategy of OD and OC, we analyzed the training dynamics. As illustrated in Supplementary Figure S1, despite the inherent class imbalance (OC is significantly smaller than OD), both loss curves decrease smoothly and converge simultaneously. This indicates that the model is not dominated by the larger OD region and effectively optimizes both structures cohesively.

## Experiment setup

### Datasets

The proposed model was evaluated on four public datasets: Drishti-GS, RIM-ONE-r3, ORIGA, and REFUGE. The DRISHTI-GS dataset [32] comprises 101 fundus images, including 70 glaucomatous and 31 normal cases. The RIM-ONE-r3 dataset [33] has 159 high-quality color fundus images. Eighty-five of them are from people without glaucoma, and 74 are from patients with glaucoma. The ORIGA dataset [34] is dedicated to glaucoma assessment and contains 650 color fundus images, of which 168 are glaucoma and 482 are normal. The REFUGE dataset [35] contains 1200 color fundus images, including 120 glaucoma cases and 1080 normal cases. All images include the labels marked by hand for the OD and OC, which were delineated by a panel of clinical experts. The experiments were run on four open-access datasets to benchmark our method. To assess domain generalization performance, we use our method under a zero-shot cross-domain setting. In this setting, we train the model on REFUGE images and directly test it on DRISHTI-GS images. Table 1 lists exactly how the data was split among training, validation, and test groups.

**Table 1:** Dataset split configuration

Dataset	Image Resolution	Train/Test
DRISHTI-GS	(1741–1845) × (2046–2468)	50/51
ORIGA	3072 × 2048	325/325
RIM-ONE-r3	2144 × 1424	127/32
REFUGE	2124 × 2056, 1634 × 1634	800/400
Cross-domain dataset	2124 × 2056, 1634 × 1634	800/51

To ensure rigorous evaluation and avoid data leakage, we adopted a two-stage training strategy. First, we performed 5-fold cross-validation (CV) on the training set to verify training stability. We reported the average metrics across the 5 folds as mean ± standard deviation, along with their 95% confidence intervals (CIs) estimated from the fold-wise performance distribution. Then, we trained the final model on the full training set and evaluated it on the held-out test set. This protocol ensures that the test set remains an unbiased benchmark for assessing final generalization performance.

### Data augmentation

Due to the limited sample size typical of medical datasets, models are prone to overfitting, which compromises their generalization capability. To enhance model robustness, we used different ways to augment the training data. The techniques applied include random horizontal flipping, random vertical flipping, random rotation, Gaussian noise injection, elastic deformation, RandStainAug, and CutMix. This augmentation pipeline tripled the training sample size.

### Evaluation indices

The model was evaluated using widely adopted metrics in image segmentation: Dice coefficient (DC), Intersection over Union (IoU). In our ablation and comparison experiments, we use the Hausdorff distance (HD) to assess how the proposed module improves boundary learning. The HD computes the maximum Euclidean distance between the predicted boundary and the ground truth boundary. A smaller value indicates more accurate boundary localization. Since this metric is sensitive to outliers, we employ the 95th percentile formulation (HD95) to lessen the effect of outliers on the evaluation. Their formulas are shown below. In these equations, TP, TN, FP, and FN denote the counts of true positive, true negative, false positive, and false negative pixels, respectively. The value of DC and IoU stay within 0 and 1. If the value is near 1, it means the model is working well.

8 $$DC{\rm{}} = {\rm{}}{{2 \times {\rm{TP}}} \over {2 \times {\rm{TP}} + {\rm{FP}} + {\rm{FN}}}}$$

9 $$IoU = {\rm{}}{{{\rm{TP}}} \over {{\rm{TP}} + {\rm{FP}} + {\rm{FN}}}}$$

10 $$HD\left( {A,B} \right) = max\left( {\mathop {\max }\limits_{a \in A} \mathop {\min }\limits_{b \in B} \parallel a - b\parallel,\mathop {\max }\limits_{b \in B} \mathop {\min }\limits_{a \in A} \parallel b - a \parallel} \right)$$

### Experiment configuration

We set up our experiments on a computing platform with Ubuntu. The setup relied on an NVIDIA GeForce RTX 3090 GPU and an Intel Xeon Platinum 8358P CPU (15 vCPUs @ 2.60 GHz). The software side ran on Python 3.8, PyTorch 1.11.0, and CUDA 12.1. During training, we chose the AdamW optimizer. The starting learning rate was 0.001, and the weight decay was set to 0.01. Each model went through 100 epochs of training. To ensure consistency, all input patches were standardized to a size of 256 × 256 × 3.

### Experiment results and analysis

We did ablation studies on four benchmark datasets. This helped us understand the contribution of each module we added. Furthermore, we tested our method against some advanced models. The findings from both numerical metrics and visual examples supports that our approach has clear advantages. To assess the agreement between our model’s cup-to-disc ratio measurements and expert annotations, we also carried out Bland-Altman and ICC analyses. These results consistently indicate that our model performs better in OD and OC segmentation.

### Ablation study

To thoroughly evaluate the efficacy of the key components, namely, EEGA module and the multi-scale loss (MSLoss) proposed in the EEGA-VMUNet architecture, we conducted ablation studies on four public fundus image datasets. Using VM-UNet as the baseline, we performed a comparative analysis of the proposed EEGA module and the original EGA module. This was achieved by incrementally integrating individual and combined components into the baseline, resulting in the following model variants: Baseline, Baseline+EGA, Baseline+EEGA(w/o GAM), Baseline+EEGA(w/o DAM), Baseline+EEGA, and Baseline+EEGA+MSLoss (i.e., EEGA-VMUNet).

We conducted a 5-fold CV ablation study on the DRISHTI-GS training set to evaluate the contribution of each component in the EEGA module. Table 2 reports the mean and standard deviation of the 5-fold CV results. The CIs are calculated based on the fold-wise results. As shown in Table 2, the baseline (VM-UNet) achieved Dice scores of 96.94% for OD and 90.42% for OC. Integrating the original EGA module and the full EEGA module into the baseline improved the OD Dice to 97.12% and 97.50%, respectively, and the OC Dice to 91.15% and 92.23%, respectively. This indicates that EEGA provides more clear performance gains over the original EGA.

**Table 2:** 5-fold CV ablation study with 95% CI on the DRISHTI-GS training set

Combination	DC(%) (95%CI)		HD95(95%CI)	Params (M)	FLOPs (G)
Baseline(VM-UNet)	Disc	96.94 ± 0.06 [96.87,97.02]	6.60 ± 0.34 [6.19, 7.04]	22.04	4.11
	Cup	90.42 ± 1.49 [88.57,92.28]	14.43 ± 2.19 [11.71, 17.15]		
Baseline+EGA	Disc	97.12 ± 0.03 [97.08,97.16]	6.56 ± 0.46 [6.00, 7.13]	29.06	7.52
	Cup	91.15 ± 1.22 [89.64,92.66]	13.04 ± 3.91 [8.18, 17.90]		
Baseline+EEGA (w/o GAM)	Disc	97.17 ± 0.02 [97.15,97.19]	6.26 ± 0.26 [5.94, 6.57]	29.28	7.71
	Cup	91.28 ± 1.18 [89.82,92.75]	12.53 ± 2.35 [9.61, 15.45]		
Baseline+EEGA (w/o DAM)	Disc	97.27 ± 0.04 [97.22,97.31]	6.11 ± 0.19 [5.88, 6.35]	30.32	8.27
	Cup	91.63 ± 0.91 [90.50,92.77]	11.40 ± 0.70 [10.53, 12.26]		
Baseline+EEGA	Disc	97.50 ± 0.10 [97.38,97.62]	5.35 ± 0.43 [4.82, 5.89]	30.56	8.47
	Cup	92.23 ± 0.94 [91.06,93.40]	10.80 ± 1.20 [9.32, 12.29]		
Baseline+EEGA+MSLoss (EEGA-VMUNet)	Disc	97.73 ± 0.13 [97.57,97.88]	5.03 ± 0.53 [4.38, 5.69]	30.56	8.47
	Cup	92.60 ± 0.84 [91.55,93.65]	9.92 ± 0.75 [8.98, 10.85]		

To further analyze the individual contributions of GAM and DAM, we removed each from the EEGA module separately. Removing GAM yielded OD/OC Dice of 97.17% and 91.28%, while removing DAM yielded 97.27% and 91.63%. Both configurations underperformed the full EEGA configuration (97.50% and 92.23%). These results verify the contribution of both branches, with GAM yielding a more evident improvement on the OC.

For the HD95 boundary metric, the baseline achieved OD/OC HD95 of 6.60 pixels and 14.43 pixels, respectively. These were reduced to 5.35 pixels and 10.80 pixels with the integration of EEGA, and further improved to 5.03 pixels and 9.92 pixels with the addition of MSLoss. The full EEGA-VMUNet configuration achieved the best results across all evaluation metrics. For the OD Dice, the 95% CI did not overlap with that of the baseline. For the OC Dice, partial overlap existed, but the interval shifted toward a more favorable direction, indicating a stable and consistent improvement. In terms of computational efficiency, the baseline has 22.04 M parameters and 4.11 G FLOPs, while the baseline+EGA has 29.06 M parameters and 7.52 G FLOPs. In comparison, the full EEGA configuration increases the parameter count to 30.56 M (+1.50 M over baseline+EGA) and FLOPs to 8.47 G (+0.95 G over baseline+EGA). Meanwhile, Baseline with EEGA improves over EGA by approximately 0.38 percentage points in OD Dice and 1.08 percentage points in OC Dice, with the HD95 reduced by approximately 2.24 pixels. Considering the performance gains brought by the EEGA module, we consider this computational cost acceptable, as accurate OD and OC boundaries facilitate subsequent CDR measurement. On the ORIGA, RIM-ONE-r3, and REFUGE datasets, we observed consistent improvement trends, with EEGA-VMUNet achieving the best performance on each dataset. Detailed results are provided in Supplementary Tables S1–S3.

**Fig. 4:**
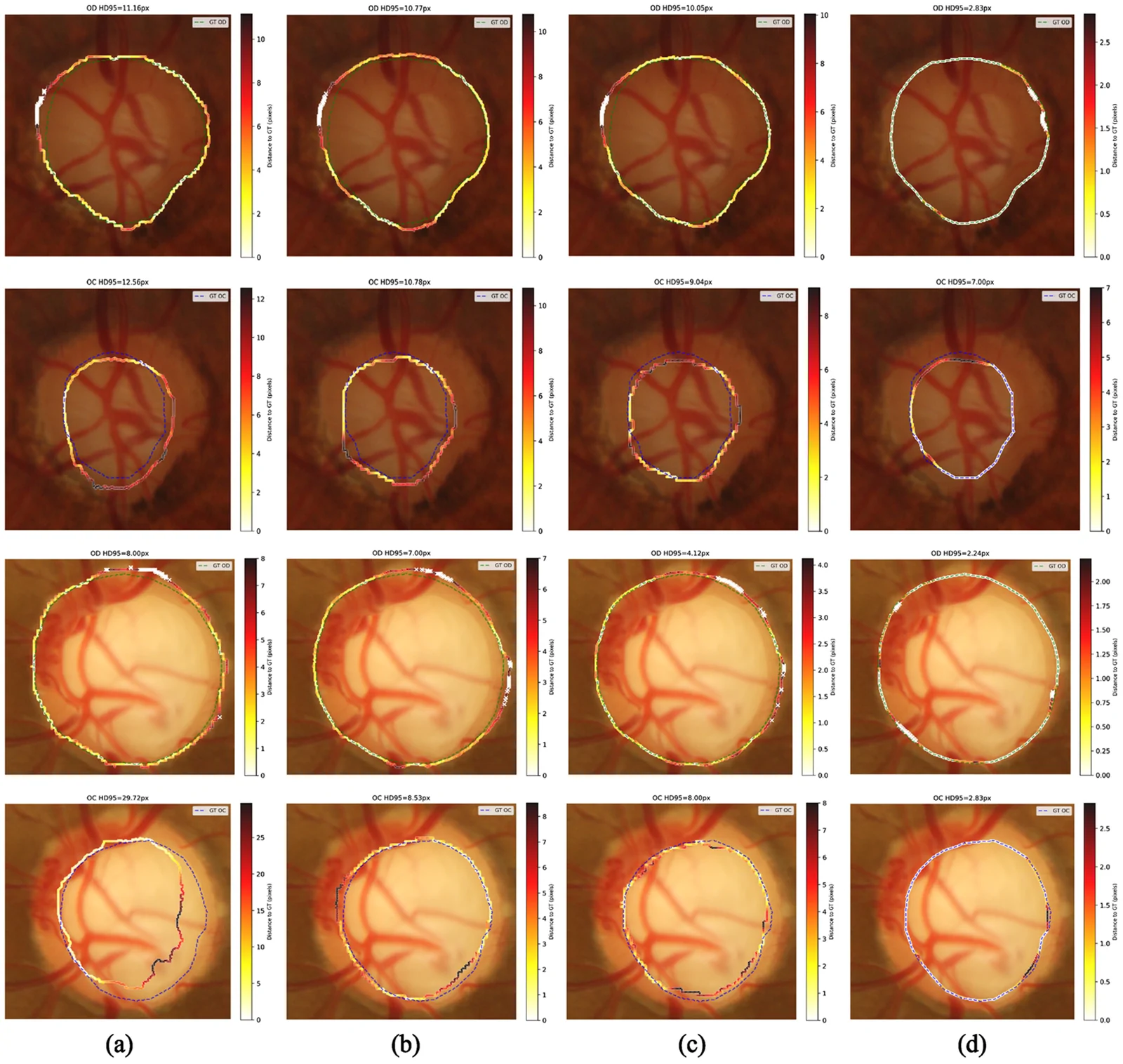
Boundary error heatmaps of different ablation variants. (**a**) Baseline, (**b**) Baseline+EGA, (**c**) Baseline+EEGA, and (**d**) Baseline+EEGA+MSLoss

Figure 4 displays boundary error heatmaps of different methods. The blue dashed lines represent the ground truth boundaries. The colorbar ranges from white through yellow and red to black, where darker colors indicate larger distances from the predicted boundary to the ground truth. The baseline method shows large dark red and black regions on both OD and OC boundaries. With the addition of EGA, the dark regions are somewhat reduced. The EEGA module further reduces the dark regions, especially on OC boundaries. Our full model produces mostly light-colored regions with fewer dark areas and achieves the lowest HD95 values. These findings collectively attest to the superior feature extraction and discriminative power of the proposed module. As a result, this helps improve segmentation accuracy for both the OD and OC.

### Comparative experiment

To comprehensively evaluate the robustness of the proposed method, we conducted a 5-fold CV on the training sets. We compare our approach against the following models: UNet [23], AGNet [36], VM-UNet [24], DA-TransUNet [31], MEGANet [28], Attention UNet with residual mechanism (denoted as Res-AttUNet) [37], EE-TransUNet [38], MobileNetV3-UNet [39], Swin-UMamba [40] and MedSAM [41]. Notably, MedSAM was evaluated using zero-shot inference only in the test set. It serves as a reference baseline for general-purpose medical segmentation models on the optic disc and cup segmentation task. Due to space constraints, we present the CV results of the DRISHTI-GS training set in the main text (Table 3), while the results for other datasets are provided in the Supplementary Material (Tables S4–S6). Fold-wise paired comparisons on the 5-fold CV were analyzed using paired t-test or Wilcoxon signed-rank test based on Shapiro-Wilk normality results. In Table 3, the statistical significance of the paired tests calculated across each fold is indicated by asterisks (*), representing *p* < 0.0056 after Bonferroni correction. Detailed pairwise *p*-value matrices for all datasets are provided in Supplementary Tables S7–S10. For the test sets, paired bootstrap resampling (1,000 iterations) was used to estimate the 95% CIs for each model’s performance. The *p*-values were derived from the bootstrap distribution of the mean differences. Given that 11 models were compared against the reference model (10 pairwise comparisons), the threshold *p*-value < 0.005 after Bonferroni correction was considered statistically significant. The results with 95% CIs are shown in Tables 4, 5, 6, 7 and 8 and the *p*-values are presented in Tables S11–S15 of the Supplementary File.

**Table 3:** Performance comparison of different models under 5-fold cross-validation with 95% CI on the DRISHTI-GS training set

Method	DC(%) (95%CI)		IoU(%) (95%CI)	HD95(95%CI)
UNet [23]	Disc	96.79 ± 0.08* [96.69,96.89]	93.78 ± 0.16* [93.58,93.98]	6.96 ± 0.48* [6.37, 7.56]
	Cup	88.76 ± 0.99* [87.53,89.99]	79.81 ± 1.60* [77.82,81.79]	16.48 ± 4.26 [11.19, 21.77]
AGNet [36]	Disc	96.89 ± 0.06* [96.8296.97]	93.97 ± 0.12* [93.83,94.12]	7.09 ± 0.86 [6.03, 8.15]
	Cup	89.75 ± 1.84 [87.47,92.03]	81.45 ± 3.05* [77.67,85.23]	15.04 ± 4.53 [9.42, 20.66]
MobileNetV3-UNet [39]	Disc	97.14 ± 0.20* [96.89,97.39]	94.44 ± 0.38* [93.97,94.91]	6.08 ± 0.40* [5.58, 6.57]
	Cup	91.70 ± 0.88* [90.61,92.80]	84.70 ± 1.49* [82.84,86.55]	11.33 ± 0.86* [10.26, 12.41]
Swin-UMamba [40]	Disc	97.18 ± 0.08* [97.08,97.28]	94.52 ± 0.16* [94.33,94.72]	6.19 ± 0.50 [5.56, 6.81]
	Cup	91.09 ± 0.88* [90.00,92.18]	83.65 ± 1.47* [81.83,85.47]	12.72 ± 2.55 [9.56, 15.88]
VM-UNet [24]	Disc	96.94 ± 0.06* [96.87,97.02]	94.07 ± 0.11* [93.93,94.20]	6.61 ± 0.34* [6.19, 7.04]
	Cup	90.42 ± 1.49* [88.57,92.28]	82.54 ± 2.48* [79.47,85.62]	14.43 ± 2.19 [11.71, 17.15]
DA-TransUNet [31]	Disc	97.34 ± 0.23 [97.05,97.62]	94.81 ± 0.44 [94.26,95.36]	5.53 ± 0.64 [4.74, 6.32]
	Cup	90.86 ± 0.94* [89.70,92.03]	83.27 ± 1.57* [81.32,85.22]	13.19 ± 2.54 [10.04, 16.34]
MEGANet [28]	Disc	97.42 ± 0.06* [97.35,97.50]	94.98 ± 0.12* [94.83,95.13]	5.77 ± 0.40* [5.28, 6.27]
	Cup	91.85 ± 0.86 [90.28,93.23]	84.94 ± 1.40 [82.27,87.30]	11.42 ± 1.30 [9.81, 13.04]
Res-AttUNet [37]	Disc	97.21 ± 0.23* [96.92,97.49]	94.56 ± 0.43* [94.03,95.10]	6.08 ± 0.78 [5.12, 7.05]
	Cup	90.56 ± 1.03* [89.28,91.85]	82.77 ± 1.71* [80.64,84.90]	13.06 ± 1.06* [11.75, 14.37]
EE-TransUNet [38]	Disc	97.35 ± 0.25 [97.04,97.66]	94.84 ± 0.48 [94.24,95.44]	5.71 ± 0.64 [4.91, 6.50]
	Cup	91.76 ± 1.19* [90.78,92.93]	84.79 ± 2.03* [83.11,86.77]	11.28 ± 1.70 [10.54, 12.76]
EEGA-VMUNet	Disc	97.73 ± 0.13 [97.57,97.88]	95.56 ± 0.24 [95.26,95.85]	5.03 ± 0.53 [4.38, 5.69]
	Cup	92.60 ± 0.84 [91.55,93.65]	86.23 ± 1.45 [84.42,88.03]	9.92 ± 0.75 [8.98, 10.85]

Note: * indicates statistical significance compared with the proposed EEGA-VMUNet, as determined by fold-wise paired tests with Bonferroni correction (p < 0.0056)

**Table 4:** Performance comparison of different models with 95% CI on the DRISHTI-GS test set

Method		DC(%) (95%CI)	IoU(%) (95%CI)	HD95(95%CI)
UNet [23]	Disc	96.68*[96.27, 97.04]	93.60*[92.91, 94.27]	7.54*[6.50, 8.74]
	Cup	85.97*[82.39, 88.90]	76.87*[72.49, 80.35]	15.46*[13.32,18.33]
AGNet [36]	Disc	96.75*[96.20, 97.24]	93.78*[92.77, 94.64]	8.41*[6.72, 10.63]
	Cup	86.32*[82.93, 89.44]	77.46*[73.36, 81.16]	15.51*[13.61, 17.82]
VM-UNet [24]	Disc	96.90*[96.53, 97.22]	94.01*[93.29, 94.63]	6.92*[6.06, 7.93]
	Cup	87.69*[84.18, 90.47]	79.58*[75.16, 83.26]	14.94[11.95, 19.32]
MobileNetV3-UNet [39]	Disc	97.01*[96.54, 97.38]	94.24*[93.36, 94.94]	6.98*[5.83, 8.36]
	Cup	88.60[85.23, 91.13]	80.71[76.72, 83.95]	12.23[10.99, 13.54]
MedSAM [41]	Disc	84.56*[81.32, 87.18]	74.56*[70.43, 78.08]	14.42*[11.41,17.59]
	Cup	82.61*[80.98, 84.36]	70.85*[68.33, 73.31]	11.85[10.10, 13.04]
Swin-UMamba [40]	Disc	97.17*[96.71, 97.55]	94.55*[93.71, 95.21]	6.88*[5.71, 8.30]
	Cup	88.72[85.86, 91.13]	80.85[77.19, 83.89]	12.62[11.14, 14.34]
DA-TransUNet [31]	Disc	97.03*[96.55, 97.44]	94.29*[93.35, 95.04]	7.00*[5.91, 8.49]
	Cup	88.64[85.86,90.84]	80.54[77.15, 83.46]	12.56[11.16, 14.07]
MEGANet [28]	Disc	97.37*[96.98, 97.69]	94.90*[94.20, 95.49]	6.17*[5.26, 7.25]
	Cup	89.44[86.35, 91.78]	82.15[77.90, 85.35]	11.66[10.11, 13.68]
Res-AttUNet [37]	Disc	96.98*[96.61, 97.29]	94.16*[93.44, 94.74]	7.02*[6.13, 8.01]
	Cup	88.75[85.91, 90.85]	80.69[77.22, 83.69]	12.16[10.73, 13.80]
EE-TransUNet [38]	Disc	97.30*[96.93, 97.60]	94.77*[94.07, 95.38]	6.46*[5.50, 7.53]
	Cup	89.32[86.17, 91.41]	81.68[78.49, 84.53]	11.88[10.46,13.42]
EEGA-VMUNet	Disc	97.65[97.31, 97.94]	95.43[94.83, 95.97]	5.03[4.45, 5.68]
	Cup	89.95[86.83, 92.18]	82.84[79.02, 85.63]	11.05[9.88, 12.30]

Note: *Indicates statistical significance (p < 0.005) compared with the proposed method, as determined by pairwise Bootstrap tests

**Table 5:** Performance comparison of different models with 95% CI on the ORIGA test set

Method		DC(%) (95%CI)	IoU(%) (95%CI)	HD95(95%CI)
UNet [23]	Disc	94.00*[93.81, 94.19]	88.73*[88.40, 89.0]	27.73*[27.02,28.40]
	Cup	86.87*[86.07, 87.61]	77.47*[76.34, 78.5]	9.22*[8.71, 9.73]
AGNet [36]	Disc	94.45*[94.25, 94.63]	89.53*[89.17,89.86]	27.43*[26.71, 28.14]
	Cup	86.88*[86.12, 87.67]	77.51*[76.32, 78.65]	8.96[8.48, 9.44]
VM-UNet [24]	Disc	94.53*[94.33, 94.72]	89.68*[89.31, 90.04]	27.67*[26.94, 28.33]
	Cup	87.20[86.49, 87.98]	77.94[76.83, 79.07]	8.86[8.41, 9.40]
MobileNetV3-UNet [39]	Disc	94.54*[94.36, 94.71]	89.68*[89.37, 89.98]	27.24*[26.61, 27.95]
	Cup	87.23[86.42, 87.99]	77.97[76.86, 79.14]	8.98[8.50, 9.42]
MedSAM [41]	Disc	87.51*[86.88, 88.10]	78.18*[77.24, 78.96]	12.81[10.97, 14.62]
	Cup	81.92*[81.23, 82.61]	69.88*[68.96, 70.85]	7.00[6.60, 7.39]
Swin-UMamba [40]	Disc	94.86*[94.67, 95.04]	90.26*[89.97, 90.55]	26.81*[26.09, 27.48]
	Cup	87.42[86.63, 88.15]	78.27[77.19, 79.30]	9.08*[8.59, 9.54]
DA-TransUNet [31]	Disc	94.76*[94.56, 94.96]	90.10*[89.74, 90.42]	27.02*[26.26, 27.72]
	Cup	87.53[86.77, 88.26]	78.45[77.36, 79.52]	8.72[8.27, 9.17]
MEGANet [28]	Disc	95.03[94.84, 95.21]	90.58[90.20, 90.93]	27.53*[26.82, 28.24]
	Cup	87.77[87.08, 88.51]	78.78[77.76, 79.85]	8.77[8.29, 9.25]
Res-AttUNet [37]	Disc	94.61*[94.43, 94.80]	89.82*[89.49, 90.14]	27.22*[26.49, 27.91]
	Cup	87.30[86.53, 88.01]	78.10[77.00, 79.18]	9.11[8.60, 9.59]
EE-TransUNet [38]	Disc	94.95*[94.78, 95.09]	90.42*[90.13, 90.71]	27.03*[26.30, 27.79]
	Cup	87.63[86.85, 88.37]	78.57[77.45, 79.59]	8.99[8.50, 9.51]
EEGA-VMUNet	Disc	95.20[95.02, 95.36]	90.88[90.60, 91.16]	26.69[25.60, 27.40]
	Cup	87.86[87.14, 88.55]	78.99[77.86, 80.11]	8.67[8.25, 9.14]

Note: *Indicates statistical significance (p < 0.005) compared with the proposed method, as determined by pairwise Bootstrap tests

**Table 6:** Performance comparison of different models with 95% CI on the RIM-ONE-r3 test set

Method	DC(%) (95%CI)		IoU(%) (95%CI)	HD95(95%CI)
UNet [23]	Disc	96.34*[95.90, 96.72]	92.98*[92.22, 93.63]	4.91*[4.43, 5.43]
	Cup	84.27*[81.51, 86.66]	74.07*[70.87, 77.41]	9.67*[8.50, 10.99]
AGNet [36]	Disc	96.45*[96.10, 96.79]	93.18*[92.47, 93.79]	4.67*[4.24, 5.08]
	Cup	85.23*[82.20, 88.03]	75.76*[72.06, 79.18]	8.81*[7.49, 10.44]
VM-UNet [24]	Disc	96.33*[95.88, 96.70]	92.97*[92.19, 93.70]	4.69*[4.28, 5.09]
	Cup	85.76*[82.79, 88.23]	76.41*[72.51, 79.77]	8.64*[7.22, 10.18]
MobileNetV3-UNet [39]	Disc	96.71*[96.29, 97.09]	93.67*[92.94, 94.40]	4.30*[3.83, 4.77]
	Cup	86.69*[83.92, 89.09]	77.80*[74.36, 80.98]	8.43*[7.37, 9.63]
MedSAM [41]	Disc	81.94*[79.96, 83.72]	70.12*[67.56, 72.85]	12.81*[10.97,14.62]
	Cup	76.14*[74.05, 77.97]	62.16*[59.53, 64.70]	8.56[7.53, 9.79]
Swin-UMamba [40]	Disc	96.95[96.59, 97.26]	94.12[93.46, 94.70]	4.06[3.70, 4.41]
	Cup	88.58[86.38, 90.52]	80.41[77.13, 83.25]	7.06[6.02, 8.55]
DA-TransUNet [31]	Disc	96.92[96.60, 97.25]	94.06[93.42, 94.74]	4.14[3.76, 4.50]
	Cup	88.31[86.20, 90.15]	79.90[76.72, 82.52]	7.45[6.57, 8.42]
MEGANet [28]	Disc	97.03[96.68, 97.35]	94.26[93.62, 94.86]	3.83[3.47, 4.23]
	Cup	88.55[86.12, 90.57]	80.42[77.30, 83.28]	6.91[6.05, 7.79]
Res-AttUNet [37]	Disc	96.74*[96.36, 97.11]	93.72*[92.98, 94.37]	4.33*[3.92, 4.75]
	Cup	85.81*[82.78, 88.37]	76.62*[72.95, 79.89]	8.49*[7.13, 10.08]
EE-TransUNet [38]	Disc	97.09[96.75, 97.42]	94.38[93.73, 95.01]	3.89[3.48, 4.33]
	Cup	88.83[86.53, 90.82]	80.92[77.62, 83.79]	6.91[5.85, 8.17]
EEGA-VMUNet	Disc	97.26[96.97, 97.54]	94.69[94.09, 95.26]	3.72[3.39, 4.07]
	Cup	89.38[86.09, 91.98]	82.38[78.41, 85.91]	6.59[5.34, 8.00]

Note: *indicates statistical significance (*p*<0.005) compared with the proposed method, as determined by pairwisebootstrap tests

**Table 7:** Performance comparison of different models with 95% CI on the REFUGE test set

Method	DC(%) (95%CI)		IoU(%) (95%CI)	HD95(95%CI)
UNet [23]	Disc	95.95*[95.72, 96.18]	92.31*[91.90, 92.72]	5.11*[4.83, 5.42]
	Cup	86.37*[85.54, 87.10]	76.71*[75.65, 77.69]	7.79*[7.37, 8.30]
AGNet [36]	Disc	96.08*[95.89, 96.27]	92.52*[92.15, 92.89]	5.90*[5.18, 6.81]
	Cup	86.85*[86.15, 87.44]	77.35*[76.36, 78.41]	7.30*[6.99, 7.65]
VM-UNet [24]	Disc	96.12*[95.89, 96.34]	92.62*[92.20, 93.04]	4.47*[4.26, 4.72]
	Cup	86.90*[86.13, 87.58]	77.47*[76.47, 78.41]	7.34*[6.96, 7.78]
MobileNetV3-UNet [39]	Disc	96.06*[95.83, 96.29]	92.51*[92.15, 92.91]	4.56*[4.35, 4.79]
	Cup	85.64*[84.88, 86.41]	75.63*[74.61, 76.69]	8.24*[7.92, 8.58]
MedSAM [41]	Disc	82.30*[81.56, 82.99]	70.58*[69.53, 71.57]	10.34*[9.86, 10.86]
	Cup	75.87*[75.15, 76.59]	61.69*[60.76, 62.64]	7.78*[7.49, 8.12]
Swin-UMamba [40]	Disc	96.39*[96.19, 96.59]	93.12*[92.69, 93.47]	4.73*[4.26, 5.31]
	Cup	87.12[86.44, 87.74]	77.76[76.80, 78.71]	7.19*[6.88, 7.53]
DA-TransUNet [31]	Disc	96.26*[96.06, 96.44]	92.85*[92.49, 93.17]	4.38*[4.19, 4.60]
	Cup	87.14*[86.46, 87.80]	77.79*[76.90, 78.72]	7.22*[6.81, 7.63]
MEGANet [28]	Disc	96.55*[96.35, 96.73]	93.40*[93.05, 93.75]	4.28*[3.96, 4.76]
	Cup	87.40[86.76, 88.04]	78.18[77.22, 79.07]	7.05[6.76, 7.35]
Res-AttUNet [37]	Disc	96.14*[95.90, 96.38]	92.68*[92.24, 93.08]	4.31*[4.10, 4.53]
	Cup	86.87*[86.17, 87.54]	77.42* [76.39, 78.43]	7.87*[7.23, 8.66]
EE-TransUNet [38]	Disc	96.43*[96.22, 96.64]	93.19*[92.81, 93.55]	4.17*[3.96, 4.40]
	Cup	87.16[86.49, 87.82]	77.79[76.82, 78.69]	7.47*[6.97, 8.19]
EEGA-VMUNet	Disc	96.92[96.76, 97.07]	94.07[93.79, 94.38]	3.57[3.41, 3.73]
	Cup	87.51[86.85, 88.10]	78.35[77.40, 79.27]	6.92[6.58, 7.25]

Note: *indicates statistical significance (*p*<0.005) compared with the proposed method, as determined by pairwisebootstrap tests

**Table 8:** Performance comparison of different models with 95% CI on the cross-domain test set

Method	DC(%) (95%CI)		IoU(%) (95%CI)	HD95(95%CI)
UNet [23]	Disc	90.33*[87.23, 92.75]	83.82*[79.57, 88.28]	15.02*[11.55, 18.65]
	Cup	79.69[75.51, 83.76]	68.50[63.41, 73.55]	22.00[18.00, 26.20]
AGNet [36]	Disc	91.38*[88.54, 93.82]	85.29*[81.27, 88.59]	14.89*[11.87, 18.23]
	Cup	80.07[76.01, 83.72]	68.86[63.61, 73.49]	21.52[17.71, 25.64]
VM-UNet [24]	Disc	93.58*[91.88, 95.04]	88.41*[85.57, 90.65]	12.10*[9.71, 15.16]
	Cup	81.03[76.96, 84.48]	70.01[64.99, 74.72]	20.44[16.91, 23.87]
MobileNetV3-UNet [39]	Disc	93.36*[91.44, 95.02]	88.15*[85.21, 90.51]	12.56*[10.18, 15.33]
	Cup	80.47*[77.00, 83.46]	68.70*[64.75, 72.33]	22.61*[19.76, 25.73]
MedSAM [41]	Disc	84.56*[81.32, 87.22]	74.56*[70.55, 78.04]	14.42[11.54, 17.87]
	Cup	82.61[80.98, 84.35]	70.85[68.39, 73.18]	11.05[9.50, 12.54]
Swin-UMamba [40]	Disc	94.15*[92.56, 95.41]	89.37*[86.78, 91.54]	12.12*[9.68, 14.86]
	Cup	81.65[77.13, 85.19]	70.99[66.14, 75.62]	19.83[17.02, 22.80]
DA-TransUNet [31]	Disc	93.83[92.20, 95.44]	88.97[86.18, 91.46]	11.54*[8.97, 14.25]
	Cup	82.19[78.27, 85.56]	71.61[67.10, 75.99]	20.50[16.82, 24.66]
MEGANet [28]	Disc	94.80[93.71, 95.60]	90.31[88.52, 91.82]	10.65[9.03, 12.76]
	Cup	82.45[78.85, 85.88]	72.00[67.56, 76.61]	19.05[15.85, 22.70]
Res-AttUNet [37]	Disc	93.43*[92.29, 94.35]	87.90*[85.98, 89.61]	16.91*[14.43, 19.71]
	Cup	81.70[78.00, 85.26]	70.84[66.02, 75.39]	19.37[16.21, 22.68]
EE-TransUNet [38]	Disc	94.45*[93.17, 95.36]	89.71*[87.80, 91.28]	11.03*[9.00, 13.63]
	Cup	82.15*[77.41, 86.15]	72.03[67.16, 76.54]	18.90*[16.56, 21.46]
EEGA-VMUNet	Disc	95.18[93.47, 96.42]	91.22[88.62, 93.28]	9.01[7.04, 11.45]
	Cup	85.13[81.46, 88.21]	75.69[71.16, 79.29]	15.72[13.96, 17.51]

Note: *Indicates statistical significance (p < 0.005) compared with the proposed method, as determined by pairwise Bootstrap tests

In the 5-fold cross-validation on DRISHTI-GS, EEGA-VMUNet achieved the highest OD Dice of 97.73% and OD IoU of 95.56% among all compared models. In the test set comparisons, paired Bootstrap tests (*p* < 0.005) confirmed statistically significant improvements in OD Dice and IoU over MEGANet on the DRISHTI-GS and REFUGE datasets. Specifically, on DRISHTI-GS test set, the OD Dice and IoU improved by 0.28% and 0.53%, with the OD HD95 reduced by 1.14 pixels. On REFUGE test set, the OD Dice and IoU improved by 0.37% and 0.67%, with the OD HD95 reduced by 0.71 pixels. For the OC, the improvements over MEGANet were relatively larger on DRISHTI-GS and RIM-ONE-r3, with increases of 0.51% and 0.83% in Dice, respectively. On RIM-ONE-r3, the model achieved an OD Dice of 97.26%, an OD IoU of 94.69%, and an OD HD95 of 3.72 pixels, numerically outperforming EE-TransUNe. On ORIGA, the model achieved an OD Dice of 95.20%, reaching a competitive performance level compared to MEGANet (95.03%).

The cross-domain test set further demonstrated the model’s robustness. The OD Dice and IoU were 95.18% and 91.22%, numerically outperforming MEGANet (94.80% and 90.31%), with the OD HD95 reduced by 1.64 pixels. In the cross-domain setting, the OC Dice and IoU improved by 2.68% and 3.69% over MEGANet, respectively. The improvements were higher than the gains observed on the same-domain datasets. Furthermore, under zero-shot inference, MedSAM achieved limited performance with an OD Dice of only 84.56% on the DRISHTI-GS test set. Overall, EEGA-VMUNet demonstrated stable and comprehensive performance across various same-domain benchmarks and cross-domain scenarios.

**Fig. 5:**
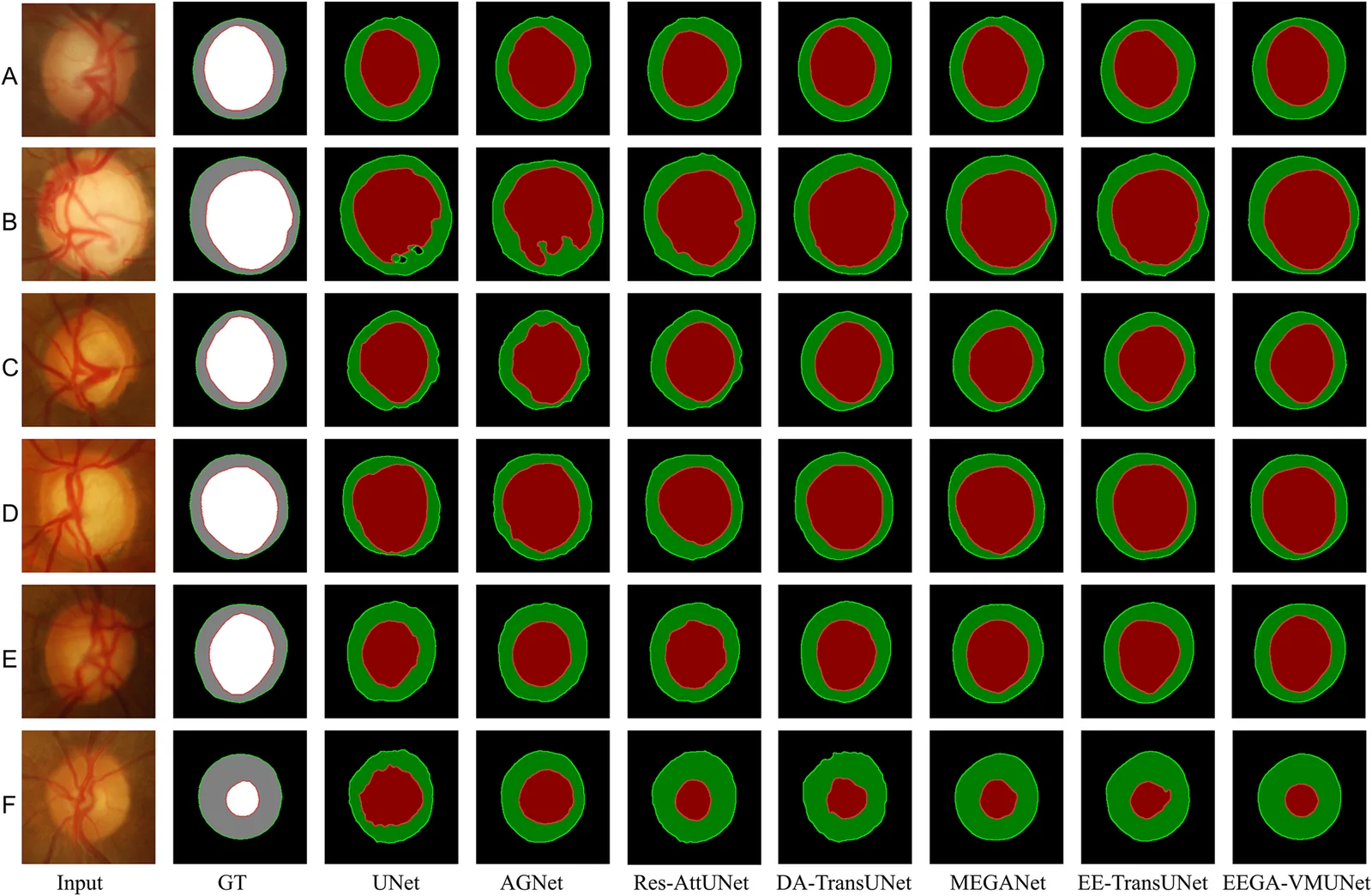
Visualization results of different models on the DRISHTI-GS dataset

**Fig. 6:**
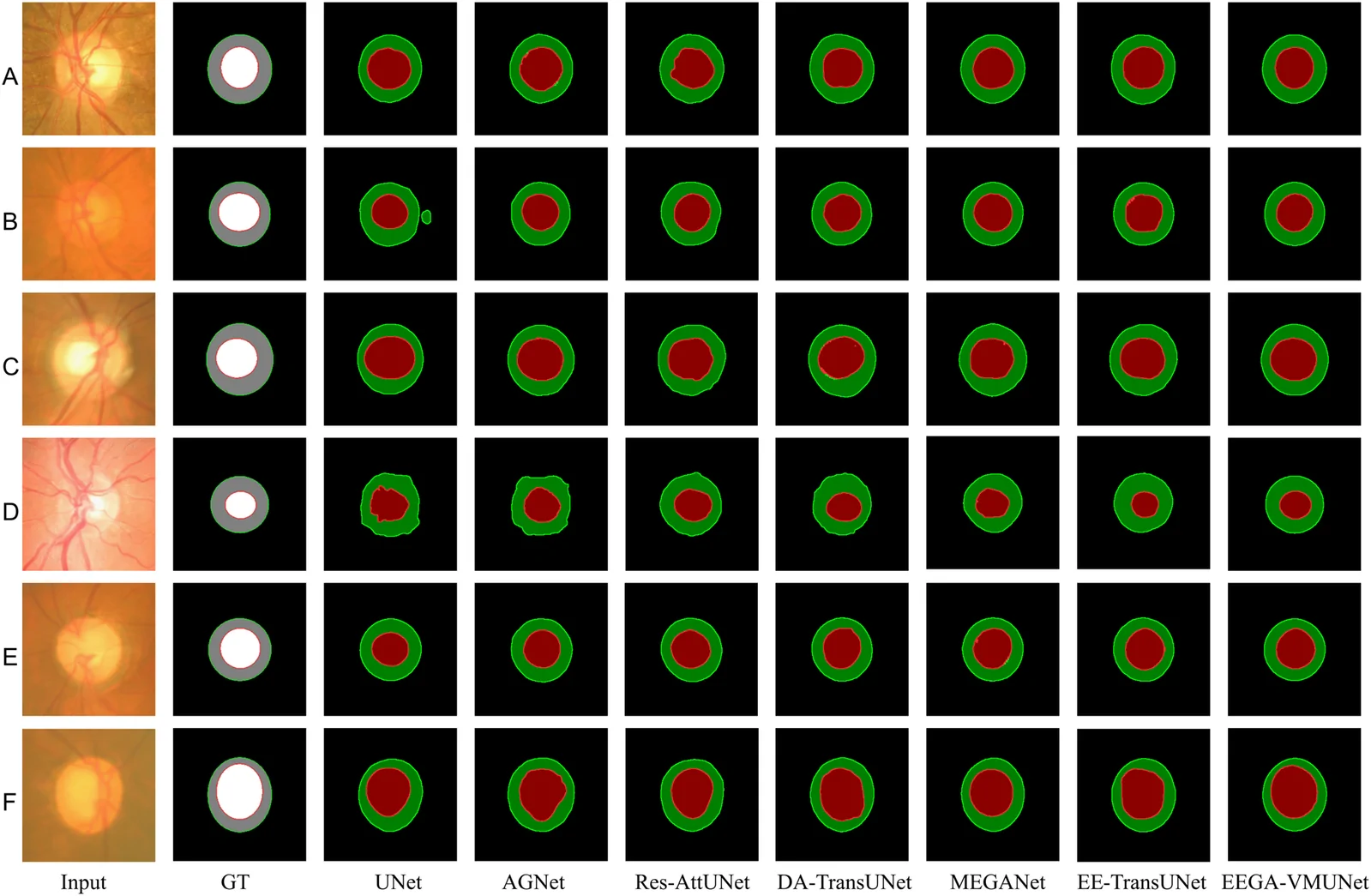
Visualization results of different models on the ORIGA dataset

**Fig. 7:**
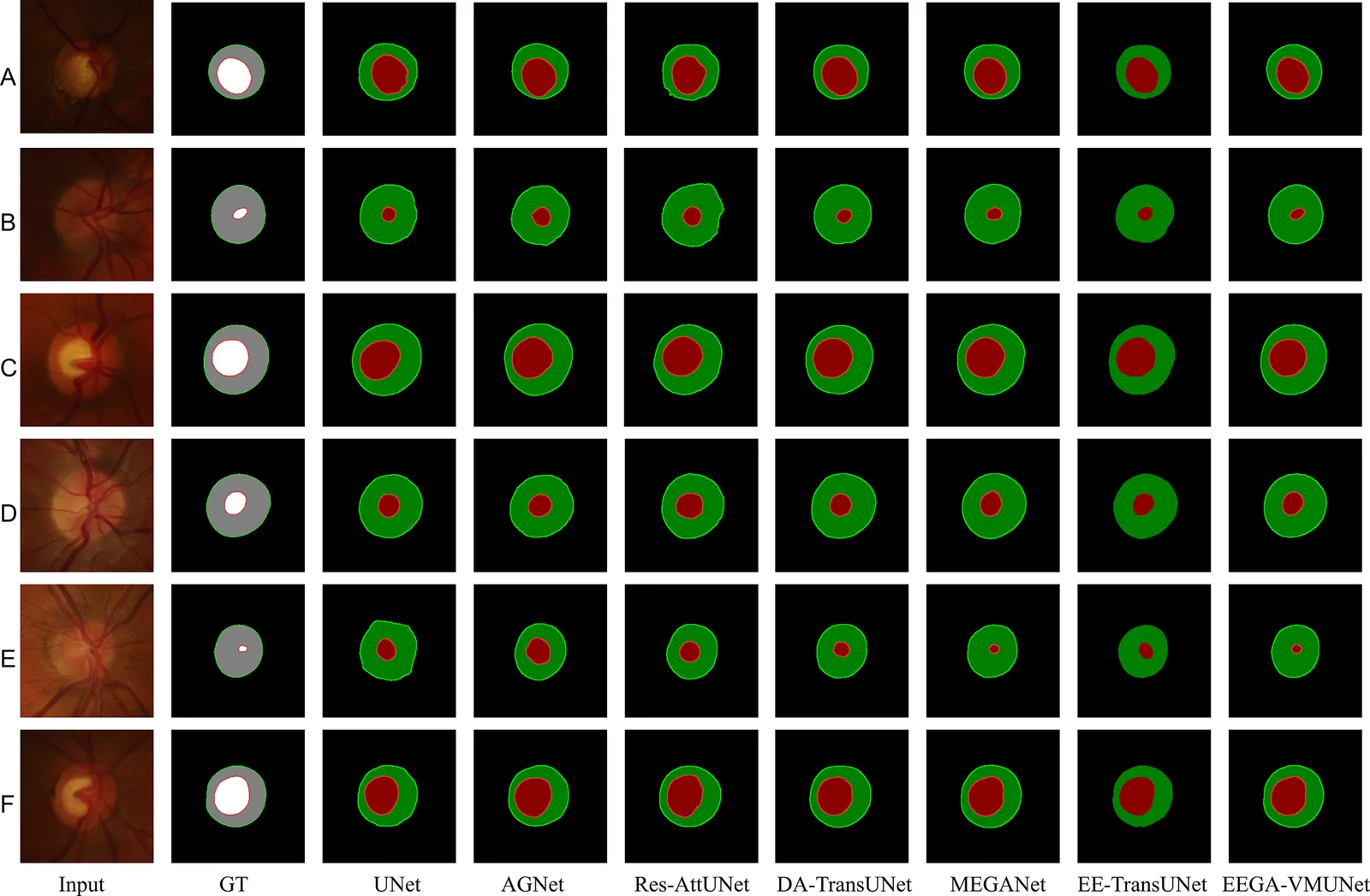
Visualization results of different models on the RIM-ONE-r3 dataset

Figure 5, 6, 7, 8 present visual comparisons of the segmentation results for the OC and OD obtained by different methods. Visually, the segmentation outputs of our proposed model most closely align with the expert-annotated ground truth. In addition to segmentation quality, we compared the computational efficiency of the models. As shown in Table 9, our model demonstrates competitive performance in terms of FLOPs, model parameters, and inference speed. This suggests the efficiency advantages of our model over advanced models, such as transformer-based architectures.

**Fig. 8:**
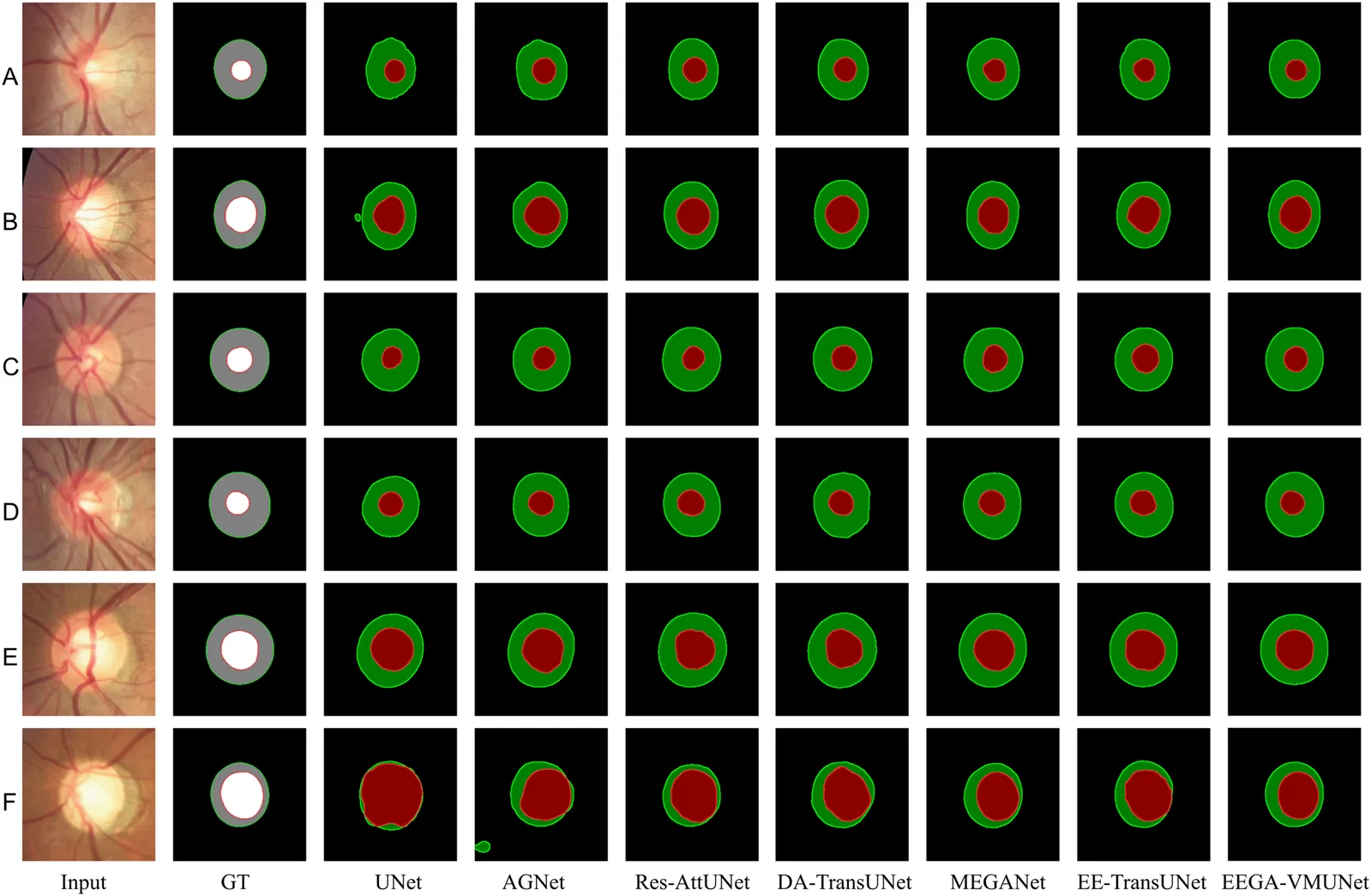
Visualization results of different models on the REFUGE dataset

**Table 9:** Comparison of computational efficiency

Method	FLOPs (G)	Parameters (M)	Inference Time (ms)
Res-AttUNet [37]	62.86	44.66	22.42
MEGANet [28]	17.05	44.89	43.99
Swin-UMamba [40]	48.90	27.39	24.75
DA-TransUNet [31]	16.87	45.46	32.24
EE-TransUNet [38]	27.77	87.99	34.73
EEGA-VMUNet	8.47	30.56	22.33

### Qualitative analysis

Fundus images vary greatly in anatomy and image quality. The OD and OC boundaries are often blurred by low contrast or retinal vessels. As shown in Figs. 5–8, the proposed EEGA-VMUNet model generates segmentation boundaries with a relatively smooth and continuous appearance. It maintains reasonable segmentation performance when processing challenging cases. For instance, in Fig. 6(A), which presents a glaucoma case with obvious vascular overlap, the model produces segmentation results that are closest to the ground truth among all compared methods. Similarly, Fig. 8(D) and 8(E) show cases with varying degrees of vessel interference. The model still yields reasonable results. In Figs. 6(E), 7(A), and 7 (B), where the image suffers from low contrast or blurred edges, the model provides adequate identification of the OD and OC region with limited boundary leakage. In Fig. 8(B), which contains a glaucoma case with peripapillary atrophy, the model still remains relatively smooth and complete. These qualitative observations are consistent with the quantitative results and suggest that our method can offer some robustness under challenging imaging conditions. This performance can be attributed to the synergistic operation of the GAM and DAM within the EEGA block.

Their integration ensures accurate boundary localization and smooth segmentation outcomes. The multi-scale loss function also enhances model performance by applying supervisory signals to feature maps at different network depths. This approach provides coarse-to-fine gradient guidance by computing losses at both the final output layer and multiple intermediate stages. Such design not only alleviates gradient vanishing but also forces the model to learn meaningful feature representations at each scale. Ultimately, this hierarchical supervision mechanism enables effective integration of information from local details to global context, ensuring segmentation accuracy. In addition, a common challenge in hybrid designs is balancing context richness with parameter efficiency. Our solution retains multi-scale information while avoiding excessive inflation of model size according to Table 9.

### Agreement analysis for cup-to-disc ratio measurement

**Fig. 9:**
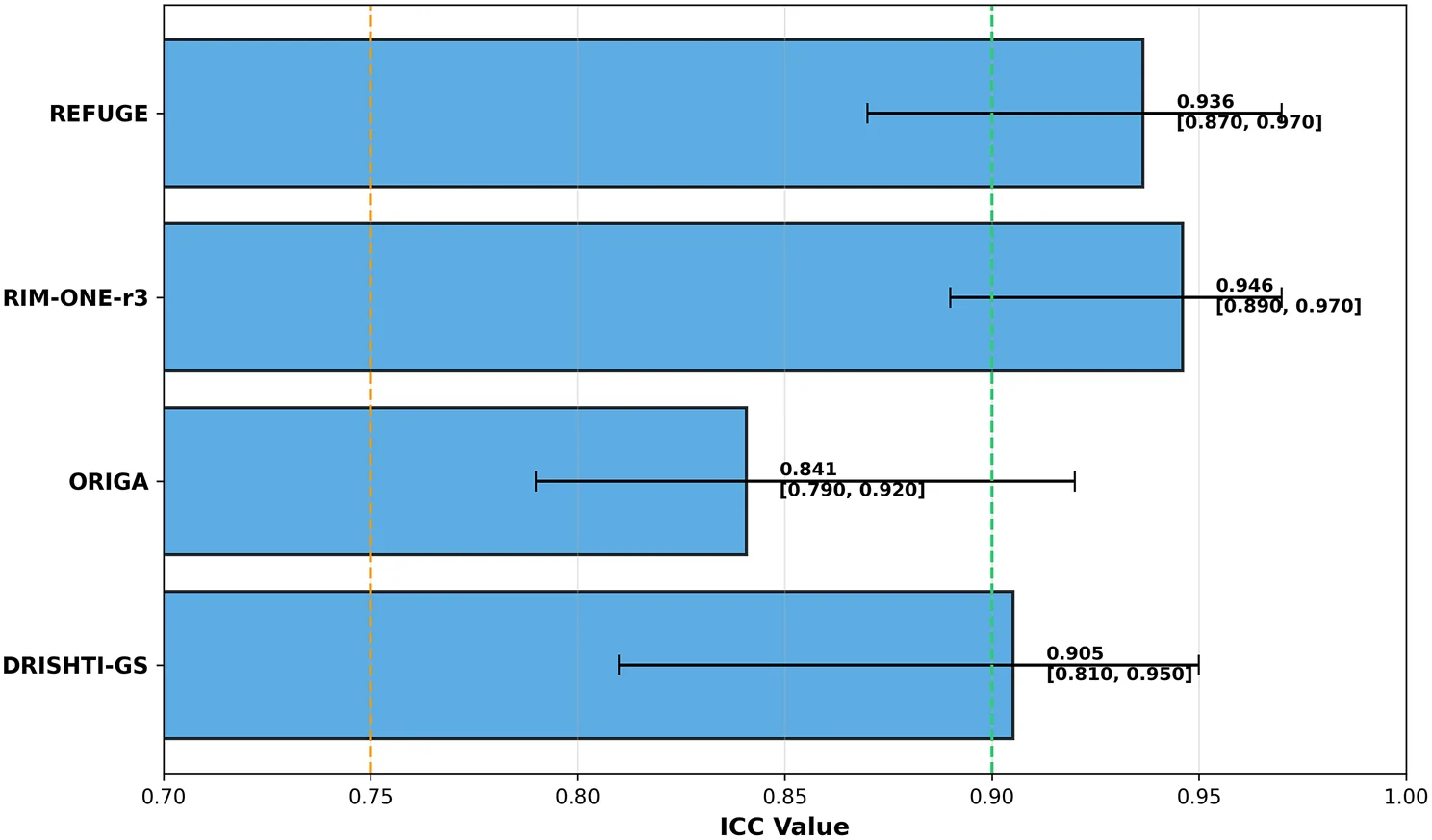
The ICC analysis results based on VCDR value across four datasets

**Fig. 10:**
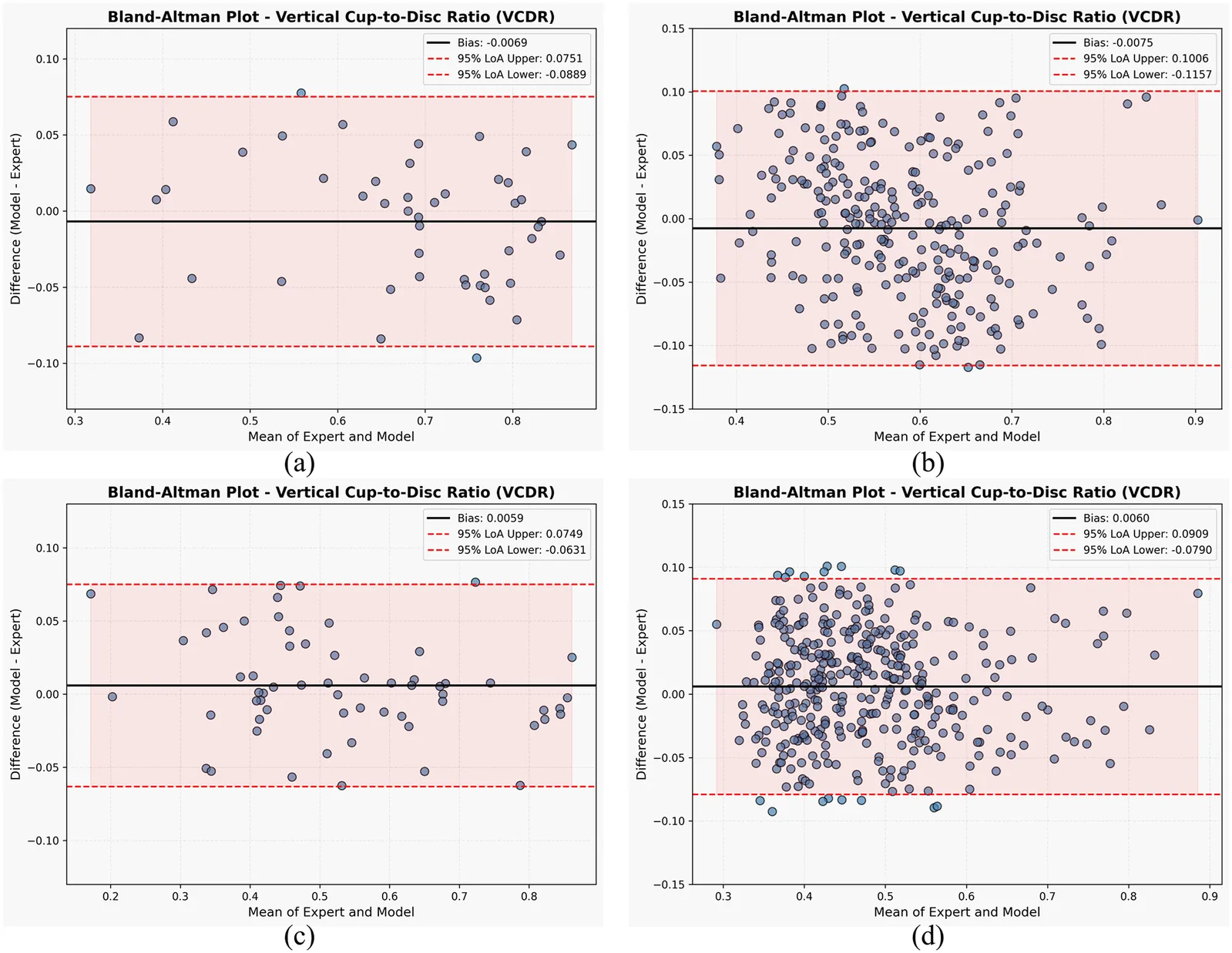
The Bland-Altman analysis results of VCDR value. (**a**) DRISHTI-GS, (**b**) ORIGA, (**c**) RIM-ONE-r3, and (**d**) REFUGE

The segmentation results of the OC and OD in fundus images can be used to calculate the VCDR value. This ratio is a key clinical sign for glaucoma screening. To check how well our model’s VCDR measurements agree with expert measurements, we ran Bland-Altman and intraclass correlation coefficient (ICC) analyses on data from four datasets. The ICC values (Fig. 9) across the four datasets were all above 0.8, ranging from 0.841 to 0.946. The Bland-Altman results are shown in Fig. 10. For DRISHTI-GS, the mean bias was −0.0069. The 95% limits of agreement ran from −0.0889 to 0.0751, and the LoA width was 0.1640. For ORIGA, the mean bias was −0.0075. Its 95% limits were −0.1157 to 0.1006, with an LoA width of 0.2163. For RIM-ONE-r3, the mean bias was 0.0059. The 95% limits ranged from −0.0631 to 0.0749, and the LoA width was 0.1380. For REFUGE, the mean bias was 0.0060. The 95% limits were −0.0790 to 0.0909, and the LoA width was 0.1699.

Across all four datasets, The absolute mean bias was below 0.01 for each one. The LoA width was under 0.22 in every case. RIM-ONE-r3 gave the narrowest LoA width, followed by DRISHTI-GS. Haslett et al. [42] found that the 95% confidence limit for inter-observer differences in CDR ratio measurements was 0.11. They also noted that a change larger than 0.1 is statistically significant. In our study, the LoA widths for the four datasets ranged from 0.1380 to 0.2163, all exceeding that benchmark. This suggests that our model achieves agreement with the expert that is broadly consistent with the trend of clinician variability reported in previous studies. However, there remains a gap between our model’s current performance and the level of clinician agreement achieved under standardized measurement conditions.

**Fig. 11:**
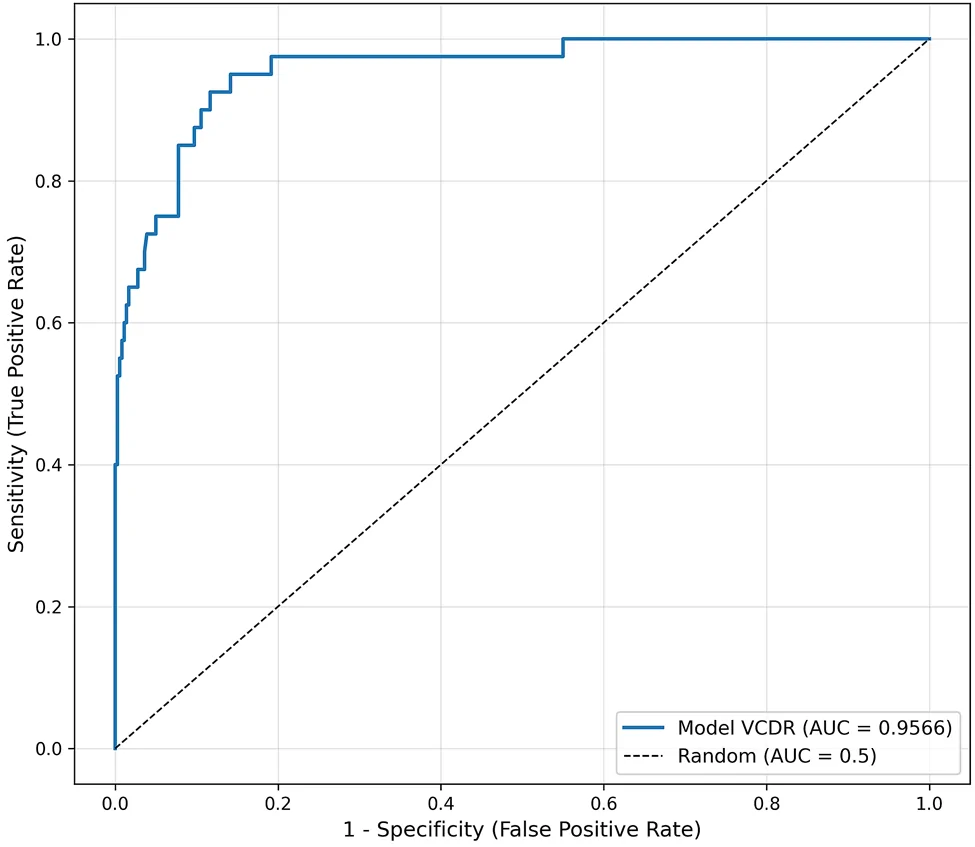
ROC curve for glaucoma identification using the model-derived VCDR on the REFUGE test set

To evaluate the screening utility of our method, we assessed its diagnostic performance against the official glaucoma labels on the REFUGE dataset (test set samples = 400). Our method achieved an AUC of 0.9566 (Fig. 11). At a VCDR threshold of 0.5, the sensitivity and specificity were 97.5% and 68.6%, respectively; at a threshold of 0.6, they were 72.5% and 95.8%, respectively.

## Discussion

In this study, we proposed EEGA-VMUNet for OD and OC segmentation. Ablation studies on the DRISHTI-GS dataset suggested that both the EEGA module and the multi-scale loss function contributed positively relative to the baseline VM-UNet. Replacing the EGA module with the proposed EEGA module improved the OC Dice coefficient from 91.15% to 92.23%. This improvement can be attributed to the dual attention design of GAM and DAM, which is intended to capture both global structural context and fine-grained boundary details. The ablation results further suggested that the two attention branches were beneficial: removing DAM from the full EEGA module decreased the OC Dice from 92.23% to 91.63%, and removing GAM decreased it to 91.28%. Introducing the multi-scale loss function further improved the metrics, raising the OC Dice from 92.23% to 92.60% and reducing the OC HD95 from 10.80 pixels to 9.92 pixels. This reduction suggests that multi-scale supervision may help the model learn more precise boundary representations. The boundary error heatmaps were consistent with this observation, showing fewer large errors in the proposed method. Similar trends were observed across the other three datasets (shown in Supplementary Tables S1–S3).

**Fig. 12:**
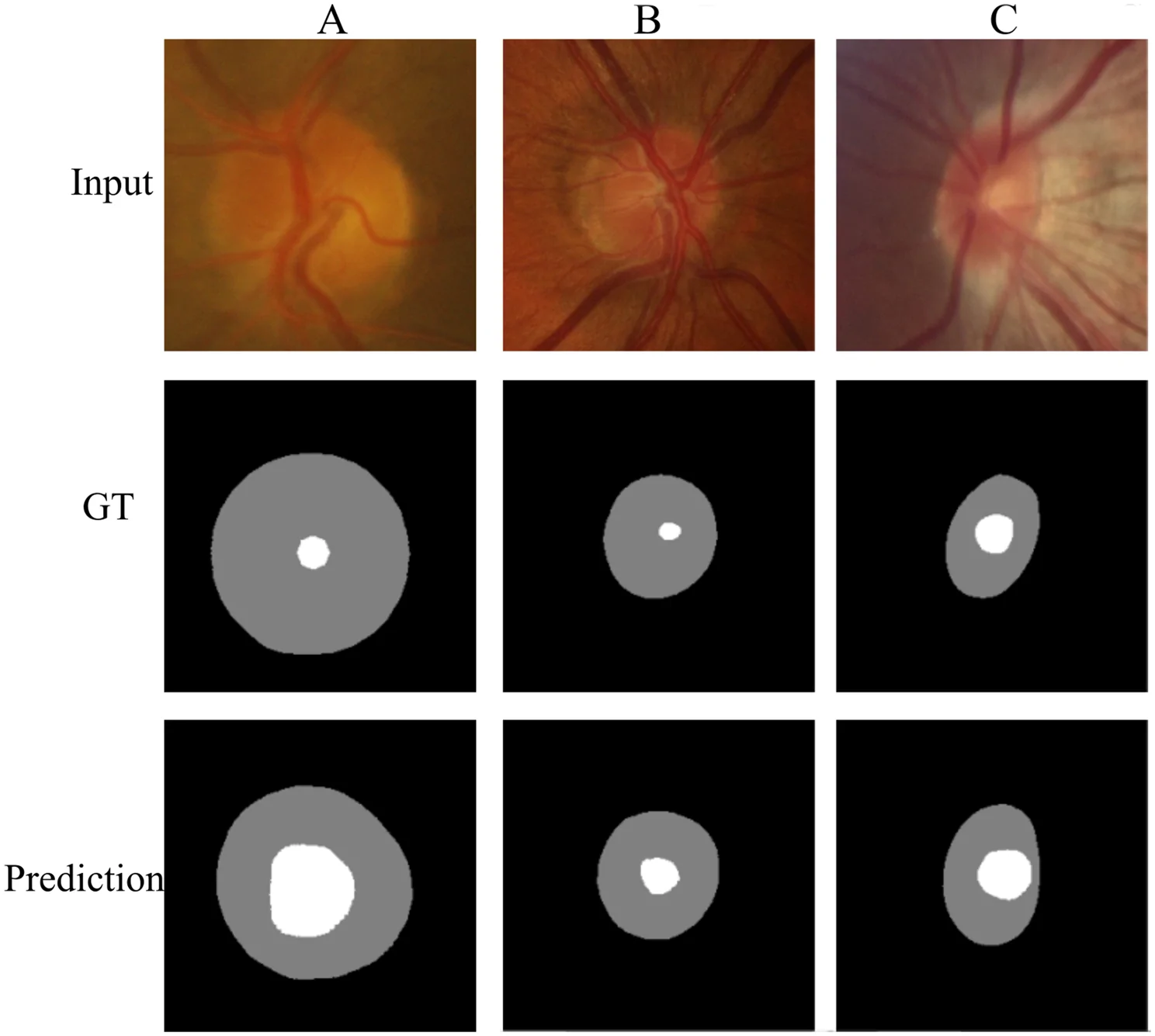
Failure cases examples

When compared with state-of-the-art models, our model achieved favorable performance. For instance, on the ORIGA dataset, it achieved Dice scores of 95.20% for the OD and 87.86% for the OC. The zero-shot cross-domain experiment further demonstrated the generalization capability of our model, achieving an OD Dice of 95.18% and an OC Dice of 85.13%. These quantitative results were corroborated by qualitative inspection of the segmentation masks, where our model produced relatively smooth and continuous boundaries. Nevertheless, we also analyzed its limitations on some challenging cases, as illustrated in Fig. 12. In cases with peripapillary atrophy (Fig. 12(A)), the model tends to over-segment the cup region, which may be attributed to the visual similarity in intensity and texture between the pathological tissue and the optic cup itself. For images with tilted discs and non-circular cup morphology (Fig. 12(C)), the model tends to generate a more symmetric cup shape, suggesting limited generalization to non-standard anatomical variations. When image quality is severely compromised, such as blurred cup margins or very low contrast (Fig. 12(B)), the model’s predictions show noticeable deviations. These observations indicate that relying solely on 2D fundus photographs may not be sufficient for highly challenging clinical scenarios. Future work could explore integrating multi-modal data, such as OCT images, or incorporating anatomical priors to further improve the model’s robustness under complex conditions.

Beyond evaluating segmentation accuracy, the clinical utility of automated CDR estimation was further assessed. We evaluated the consistency between the automated CDR measurements and expert annotations using Bland-Altman analysis. Across the four datasets, the mean bias was minimal and remained below 0.01. However, the LoA widths for the four datasets ranged from 0.1380 to 0.2163, all exceeding the minimum detectable change required for detecting early glaucomatous progression. This indicates that the current method may not be sufficiently precise for longitudinal monitoring of individual patients. In addition, our diagnostic evaluation on the REFUGE dataset showed that the model’s VCDR measurements achieved an AUC of 0.9566, with sensitivity and specificity of 97.5% and 68.6% at a CDR threshold of 0.5, and 72.5% and 95.8% at a threshold of 0.6. These results reflect the inherent trade-off between sensitivity and specificity commonly observed in diagnostic tests. The specificity at the 0.5 threshold is modest, and the sensitivity at the 0.6 threshold is limited. However, the high sensitivity at the 0.5 threshold suggests that the method may help minimize missed cases in screening settings, where detecting potential glaucoma cases is prioritized over confirming diagnoses. These results are not yet sufficient for standalone clinical diagnosis, but they indicate potential auxiliary value in large-scale screening workflows where expert resources are limited. In terms of computational efficiency, the model contains 30.56 million parameters and has less inference time than models like EE-TransUNet. Despite the inclusion of Swin-UMamba, the scope of our comparative analysis lacks a direct evaluation against other emerging Mamba-based architectures. Additionally, the YOLO-based preprocessing module in our pipeline is currently optimized for the specific characteristics of the training datasets, which may constrain its direct adaptability to other imaging modalities or resolutions without fine-tuning. While the segmentation network itself achieves favorable performance in cross-domain settings, the generality of the preprocessing pipeline remains to be further explored. In future work, we plan to investigate generalized preprocessing strategies and extend our method to more diverse Mamba variants to further validate its robustness.

## Conclusion

In this study, we proposed a hybrid framework for OD and OC segmentation in fundus images. The framework integrates a YOLO-based preprocessing module for coarse OD localization and an EEGA-VMUNet segmentation network equipped with a dual-attention EEGA module. The EEGA module adaptively aggregates multi-scale contextual information through global and detail attention branches, enhancing the representation of fine-grained boundaries. Ablation studies and comparison experiments across four datasets demonstrated that EEGA-VMUNet achieved favorable segmentation accuracy. Furthermore, the VCDR measurements derived from our segmentations showed potential as a diagnostic aid for glaucoma screening. However, several limitations remain. First, the evaluation was conducted on datasets from limited sources, and the generalizability of the YOLO-based preprocessing pipeline to other imaging modalities or resolutions requires further validation. Second, although EEGA-VMUNet offers a reasonable balance between parameter efficiency and inference speed relative to several competing models, it still requires further lightweight optimization to facilitate deployment on resource-constrained devices. Additionally, as indicated by the Bland-Altman analysis, the current precision may still be insufficient for longitudinal monitoring of individual patients. In future work, we plan to validate our method on large-scale, multi-center clinical datasets acquired from diverse imaging devices. We also intend to explore the integration of multi-modal data, such as OCT images, to improve robustness in challenging cases including peripapillary atrophy and tilted discs. Finally, model compression strategies and lightweight backbone designs will be investigated to facilitate deployment in community screening settings.

## Electronic supplementary material

Below is the link to the electronic supplementary material.

Supplementary material 1

## Data Availability

DRISHTI-GS, ORIGA, RIM-ONE-r3, and REFUGE datasets are available in Refs. [32–35].
