# Supplementary material for: Optic disc and cup segmentation using Vision Mamba UNet with enhanced edge-guided attention in fundus images
Source: BMC Ophthalmol. 2026 Oct 23;26:691. doi: 10.1186/s12886-026-05371-x (PMC13711713; doi:10.1186/s12886-026-05371-x)
Supplement: Supplementary file 1 [file 12886_2026_5371_MOESM1_ESM.docx]

**Supporting Information**

Supplementary Table S1. Comparison of 5-fold CV ablation study results with 95% CI on the ORIGA training set.

Supplementary Table S2. Comparison of 5-fold CV ablation study results with 95% CI on the RIM-ONE-r3 training set.

Supplementary Table S3. Comparison of 5-fold CV ablation study results with 95% CI on the REFUGE training set.

Supplementary Table S4. Performance comparison of different models under 5-fold cross-validation with 95% CI on the ORIGA training set.

Supplementary Table S5. Performance comparison of different models under 5-fold cross-validation with 95% CI on the RIM-ONE-r3 training set.

Supplementary Table S6. Performance comparison of different models under 5-fold cross-validation with 95% CI on the REFUGE training set.

Supplementary Table S7. P-values of paired tests between EEGA-VMUNet and competing methods across 5 folds on the DRISHTI-GS training set.

Supplementary Table S8. P-values of paired tests between EEGA-VMUNet and competing methods across 5 folds on the ORIGA training set.

Supplementary Table S9. P-values of paired tests between EEGA-VMUNet and competing methods across 5 folds on the RIM-ONE-r3 training set.

Supplementary Table S10. P-values of paired tests between EEGA-VMUNet and competing methods across 5 folds on the REFUGE training set.

Supplementary Table S11. Pairwise p-values (Bootstrap-based) for EEGA-VMUNet versus competing methods on the DRISHTI-GS test set.

Supplementary Table S12. Pairwise p-values (Bootstrap-based) for EEGA-VMUNet versus competing methods on the ORIGA test set.

Supplementary Table S13. Pairwise p-values (Bootstrap-based) for EEGA-VMUNet versus competing methods on the RIM-ONE-r3 test set.

Supplementary Table S14. Pairwise p-values (Bootstrap-based) for EEGA-VMUNet versus competing methods on the REFUGE test set.

Supplementary Table S15. Pairwise p-values (Bootstrap-based) for EEGA-VMUNet versus competing methods on the cross-domain test set.

Supplementary Figure S1. Training loss curves on different datasets.

Supplementary Table S1. Comparison of 5-fold CV ablation study results with 95% CI on the ORIGA training set.

| Combination | DC(%) (95%CI) | | IoU(%) (95%CI) | HD95(95%CI) |
| --- | --- | --- | --- | --- |
| Baseline(VM-UNet) | Disc | 94.44 ± 0.26  [94.12,94.76] | 89.46 ± 0.47  [88.88,90.04] | 23.05 ±7.66  [13.54,32.56] |
|  | Cup | 87.84 ± 0.59  [87.10,88.57] | 78.32 ± 0.94  [77.15,79.48] | 6.98 ± 2.39  [4.02,9.94] |
| Baseline+EGA | Disc | 94.66 ± 0.26  [94.34,94.98] | 89.86 ± 0.46  [89.29,90.42] | 22.75 ± 7.48  [13.46,32.03] |
|  | Cup | 88.11 ± 0.65  [87.31,88.92] | 78.75 ± 1.03  [77.48,80.03] | 6.94 ± 2.35  [4.03,9.86] |
| Baseline+EEGA  (w/o GAM) | Disc | 94.69 ± 0.25  [94.38,95.00] | 89.91 ± 0.45  [89.35,90.46] | 22.63 ±7.44  [13.39,31.87] |
|  | Cup | 88.20 ± 0.67  [87.37,89.03] | 78.90 ± 1.07  [77.58,80.22] | 6.83 ± 2.40  [3.85,9.81] |
| Baseline+EEGA  (w/o DAM) | Disc | 94.84 ± 0.29  [94.48,95.20] | 90.18 ± 0.53  [89.52,90.84] | 22.47 ±7.38  [13.32,31.63] |
|  | Cup | 88.36 ± 0.69  [87.50,89.22] | 79.15 ± 1.11  [77.77,80.53] | 6.75 ± 2.43  [3.73,9.78] |
| Baseline+EEGA | Disc | 95.05 ± 0.29  [94.70,95.41] | 90.57 ± 0.51  [89.93,91.21] | 22.22 ±7.37  [13.06,31.37] |
|  | Cup | 88.59 ± 0.73  [87.68,89.49] | 79.52 ± 1.17  [78.06,80.97] | 6.65 ± 2.49  [3.56,9.75] |
| Baseline+EEGA+MSLoss  (EEGA-VMUNet) | Disc | 95.22 ± 0.32  [94.82,95.62] | 90.87 ± 0.59  [90.14,91.61] | 22.03 ±7.27  [13.01,31.05] |
|  | Cup | 88.71 ± 0.77  [87.76,89.67] | 79.72 ± 1.24  [78.18,81.26] | 6.59 ± 2.46  [3.54,9.64] |

Supplementary Table S2. Comparison of 5-fold CV ablation study results with 95% CI on the RIM-ONE-r3 training set.

| Combination | DC(%) (95%CI) | | IoU(%) (95%CI) | HD95(95%CI) |
| --- | --- | --- | --- | --- |
| Baseline(VM-UNet) | Disc | 96.49 ± 0.16  [96.29,96.68] | 93.21 ± 0.29  [92.85,93.57] | 4.95 ± 0.64  [4.16, 5.74] |
|  | Cup | 88.01 ± 0.56  [87.32,88.70] | 78.59 ± 0.89  [77.48,79.70] | 4.89 ± 1.73  [2.75, 7.04] |
| Baseline+EGA | Disc | 96.71 ± 0.09  [96.60,96.83] | 93.64 ± 0.17  [93.43,93.84] | 4.55 ± 0.24  [4.25, 4.85] |
|  | Cup | 88.59 ± 0.64  [87.80,89.38] | 79.52 ± 1.03  [78.24,80.79] | 4.64 ± 1.75  [2.46, 6.81] |
| Baseline+EEGA  (w/o GAM) | Disc | 96.78 ± 0.08  [96.68,96.88] | 93.76 ± 0.15  [93.57,93.95] | 4.51 ± 0.23  [4.23, 4.79] |
|  | Cup | 88.69 ± 0.63  [87.91,89.48] | 79.69 ± 1.02  [78.00,81.00] | 4.59 ± 1.78  [2.39, 6.80] |
| Baseline+EEGA  (w/o DAM) | Disc | 96.85 ± 0.06  [96.77,96.92] | 93.88 ± 0.12  [93.73,94.03] | 4.37 ± 0.22  [4.10, 4.64] |
|  | Cup | 88.88 ±0.71  [88.00,89.76] | 79.99 ± 1.15  [78.56,81.41] | 4.51 ± 1.76  [2.33, 6.70] |
| Baseline+EEGA | Disc | 97.09 ± 0.07  [97.00,97.18] | 94.34 ± 0.13  [94.19,94.50] | 4.05 ± 0.20  [3.81, 4.30] |
|  | Cup | 89.44 ± 0.91  [88.31,90.58] | 80.91 ± 1.50  [79.05,82.77] | 4.40 ± 1.72  [2.27, 6.53] |
| Baseline+EEGA+MSLoss  (EEGA-VMUNet) | Disc | 97.29 ± 0.12  [97.14,97.44] | 94.73 ± 0.23  [94.45,95.01] | 3.62 ± 0.32  [3.22, 4.01] |
|  | Cup | 89.79 ± 0.93  [88.63,90.94] | 81.48 ± 1.53  [79.58,83.38] | 4.25 ± 1.61  [2.25, 6.25] |

Supplementary Table S3. Comparison of 5-fold CV ablation study results with 95% CI on the REFUGE training set.

| Combination | DC(%) (95%CI) | | IoU(%) (95%CI) | HD95(95%CI) |
| --- | --- | --- | --- | --- |
| Baseline(VM-UNet) | Disc | 96.26 ± 0.17  [96.05,96.47] | 92.79 ± 0.31  [92.41,93.18] | 6.11 ± 0.36  [5.67, 6.55] |
|  | Cup | 87.76 ± 0.50  [87.14,88.38] | 78.19 ± 0.79  [77.21,79.17] | 9.44 ± 0.57  [8.73, 10.14] |
| Baseline+EGA | Disc | 96.47 ± 0.16  [96.27,96.66] | 93.17 ± 0.30  [92.80,93.54] | 5.72 ± 0.28  [5.37, 6.07] |
|  | Cup | 88.17 ± 0.48  [87.58,88.76] | 78.85 ± 0.75  [77.91,79.79] | 9.01 ± 0.27  [8.67, 9.34] |
| Baseline+EEGA  (w/o GAM) | Disc | 96.57 ± 0.13  [96.40,96.74] | 93.37 ± 0.25  [93.06,93.68] | 5.34 ± 0.27  [5.00, 5.68] |
|  | Cup | 88.24 ± 0.47  [87.65,88.82] | 78.95 ± 0.75  [78.01,79.88] | 8.82 ± 0.28  [8.47, 9.17] |
| Baseline+EEGA  (w/o DAM) | Disc | 96.71 ± 0.14  [96.54,96.89] | 93.63 ± 0.26  [93.31,93.96] | 5.21 ± 0.33  [4.80, 5.62] |
|  | Cup | 88.41 ± 0.57  [87.71,89.11] | 79.20 ± 0.91  [78.10,80.36] | 8.64 ± 0.23  [8.36, 8.92] |
| Baseline+EEGA | Disc | 97.02 ± 0.13  [96.86,97.18] | 94.21 ± 0.24  [93.92,94.51] | 4.68 ± 0.23  [4.40, 4.97] |
|  | Cup | 88.67 ± 0.60  [87.93,89.41] | 79.65 ± 0.96  [78.45,80.85] | 8.51 ± 0.32  [8.12, 8.91] |
| Baseline+EEGA+MSLoss  (EEGA-VMUNet) | Disc | 97.21 ± 0.10  [97.09,97.32] | 94.56 ± 0.18  [94.34,94.79] | 4.40 ± 0.19  [4.17, 4.63] |
|  | Cup | 88.92 ± 0.61  [88.16,89.67] | 80.06 ± 0.98  [78.84,81.28] | 8.35± 0.30  [7.98, 8.73] |

Supplementary Table S4. Performance comparison of different models under 5-fold cross-validation with 95% CI on the ORIGA training set.

| Method | DC(%) (95%CI) | | IoU(%) (95%CI) | HD95(95%CI) |
| --- | --- | --- | --- | --- |
| UNet [23] | Disc | 94.07 ± 0.15*  [93.88,94.25] | 88.80 ± 0.27*  [88.47,89.13] | 23.39 ± 7.73  [13.79,32.99] |
|  | Cup | 87.32 ± 0.46*  [86.76,87.89] | 77.51 ± 0.72*  [76.62,78.39] | 7.40 ± 2.37  [4.46,10.34] |
| AGNet [36] | Disc | 94.28 ± 0.24*  [93.98,94.57] | 89.17 ± 0.43*  [88.64,89.70] | 23.20 ± 7.65  [13.71,32.70] |
|  | Cup | 87.67 ± 0.58*  [86.95,88.39] | 78.05 ± 0.92*  [76.91,79.19] | 7.05± 2.36  [4.12,9.98] |
| MobileNetV3-UNet [39] | Disc | 94.59 ± 0.24*  [94.29,94.89] | 89.74 ± 0.44*  [89.19,90.29] | 22.96 ± 7.64  [13.46,32.45] |
|  | Cup | 88.05 ± 0.57  [87.34,88.76] | 78.66 ± 0.91  [77.54,79.78] | 6.89 ± 2.40  [3.91,9.87] |
| Swin-UMamba [40] | Disc | 94.85 ± 0.29*  [94.48,95.21] | 90.20± 0.53*  [89.54,90.86] | 22.54 ± 7.48  [13.26,31.82] |
|  | Cup | 88.31 ± 0.73*  [87.40,89.22] | 79.07 ± 1.17*  [77.62,80.53] | 6.73 ± 2.44  [3.70,9.75] |
| VM-UNet [24] | Disc | 94.44 ± 0.26*  [94.12,94.76] | 89.46 ± 0.47*  [88.88,90.04] | 23.05 ± 7.66  [13.54,32.56] |
|  | Cup | 87.84 ± 0.59*  [87.10,88.57] | 78.32± 0.94*  [77.15,79.48] | 6.98 ± 2.39  [4.02,9.94] |
| DA-TransUNet [31] | Disc | 94.73 ± 0.30*  [94.36,95.11] | 89.99 ± 0.54*  [89.31,90.67] | 22.66 ± 7.54  [13.30,32.02] |
|  | Cup | 88.23 ± 0.70*  [87.36,89.10] | 78.95 ± 1.12*  [77.56,80.34] | 6.78 ± 2.43*  [3.76,9.80] |
| MEGANet [28] | Disc | 95.03 ± 0.28*  [94.68,95.38] | 90.53 ± 0.51*  [89.89,91.17] | 22.16 ± 7.28  [13.11,31.20] |
|  | Cup | 88.54 ± 0.81*  [87.54,89.55] | 79.45± 1.30*  [77.84,81.06] | 6.64 ± 2.48  [3.56,9.72] |
| Res-AttUNet [37] | Disc | 94.61 ± 0.24*  [94.31,94.91] | 89.77 ± 0.43*  [89.23,90.30] | 22.77 ± 7.58  [13.36,32.19] |
|  | Cup | 88.05 ± 0.59*  [87.31,88.79] | 78.65 ± 0.94*  [77.48,79.82] | 6.85 ± 2.42  [3.85,9.85] |
| EE-TransUNet [38] | Disc | 94.91 ± 0.33*  [94.51,95.32] | 90.32 ± 0.59*  [89.58,91.06] | 22.34 ± 7.37  [13.19, 31.50] |
|  | Cup | 88.37 ± 0.76*  [87.42,89.32] | 79.18 ± 1.23*  [77.65,80.70] | 6.69 ± 2.45  [3.64, 9.74] |
| EEGA-VMUNet | Disc | 95.22 ± 0.32  [94.82,95.62] | 90.87 ± 0.59  [90.14,91.61] | 22.03 ± 7.27  [13.01,31.05] |
|  | Cup | 88.71 ± 0.77  [87.76,89.67] | 79.72 ± 1.24  [78.18,81.26] | 6.59 ± 2.46  [3.54,9.64] |

**Note: * indicates statistical significance compared with the proposed EEGA-VMUNet (p<0.0056), The Shapiro-Wilk test was used to assess the normality of the data. Paired t-tests were used for normally distributed data, while Wilcoxon signed-rank tests were used otherwise. P-values were adjusted using the Bonferroni correction for multiple comparisons.**

Supplementary Table S5. Performance comparison of different models under 5-fold cross-validation with 95% CI on the RIM-ONE-r3 training set.

| Method | DC(%) (95%CI) | | IoU(%) (95%CI) | HD95(95%CI) |
| --- | --- | --- | --- | --- |
| UNet [23] | Disc | 96.29 ± 0.22*  [96.02, 96.56] | 92.84 ± 0.40*  [92.34,93.34] | 5.10 ± 0.26*  [4.78, 5.42] |
|  | Cup | 87.12 ± 0.43*  [86.58,87.65] | 77.17 ± 0.67*  [76.34,78.00] | 5.69 ± 1.77  [3.49, 7.89] |
| AGNet [36] | Disc | 96.65 ± 0.10*  [96.52,96.78] | 93.52 ± 0.19*  [93.28,93.76] | 4.80 ± 0.25  [4.50, 5.11] |
|  | Cup | 87.49 ± 0.47*  [86.91,88.08] | 77.77 ± 0.74*  [76.85,78.69] | 5.44 ± 1.69  [3.35, 7.53] |
| MobileNetV3-UNet [39] | Disc | 96.86 ± 0.06*  [96.79,96.93] | 93.92 ± 0.10*  [93.79,94.04] | 4.25 ± 0.22  [3.97, 4.53] |
|  | Cup | 88.47 ± 0.52  [87.82,89.12] | 79.33 ± 0.84  [78.29,80.37] | 4.76 ± 1.71  [2.63, 6.88] |
| Swin-UMamba [40] | Disc | 96.95 ± 0.10*  [96.82,97.07] | 94.08 ± 0.19*  [93.85,94.31] | 4.16 ± 0.24  [3.87, 4.46] |
|  | Cup | 89.04 ± 0.86*  [87.97,90.11] | 80.26 ± 1.40*  [78.51,82.00] | 4.42 ± 1.73  [2.28, 6.57] |
| VM-UNet [24] | Disc | 96.49 ± 0.16*  [96.29,96.68] | 93.21 ± 0.29*  [92.85,93.57] | 4.95 ± 0.64*  [4.16, 5.74] |
|  | Cup | 88.01 ± 0.56*  [87.32,88.70] | 78.59 ± 0.89*  [77.48,79.70] | 4.89 ± 1.73  [2.75, 7.04] |
| DA-TransUNet [31] | Disc | 96.96 ± 0.08  [96.87,97.06] | 94.11 ± 0.14  [93.93,94.28] | 4.16 ± 0.16  [3.95, 4.36] |
|  | Cup | 88.91 ± 0.81*  [87.90,89.92] | 80.04 ± 1.32*  [78.40,81.68] | 4.62 ± 1.78  [2.40, 6.83] |
| MEGANet [28] | Disc | 97.02 ± 0.06  [96.95,97.10] | 94.22 ± 0.12  [94.07,94.37] | 3.98 ± 0.13  [3.82, 4.14] |
|  | Cup | 89.32 ± 0.94*  [88.16,90.49] | 80.72 ± 1.54*  [78.81,82.63] | 4.41 ± 1.71  [2.28, 6.54] |
| Res-AttUNet [37] | Disc | 96.90 ± 0.07  [96.82,96.98] | 93.99 ± 0.12  [93.84,94.14] | 4.21 ± 0.14  [4.03, 4.38] |
|  | Cup | 88.36 ± 0.48  [87.76,88.96] | 79.15 ± 0.78  [78.18,80.12] | 4.77 ± 1.75  [2.61, 6.94] |
| EE-TransUNet [38] | Disc | 97.04 ± 0.10  [96.91,97.17] | 94.26 ± 0.19  [94.02,94.49] | 4.08 ± 0.30*  [3.70, 4.45] |
|  | Cup | 89.21 ± 0.92  [88.07,90.35] | 80.53 ± 1.51  [78.66,82.40] | 4.50 ± 1.78  [2.29, 6.71] |
| EEGA-VMUNet | Disc | 97.29 ± 0.12  [97.14,97.44] | 94.73 ±0.23  [94.45,95.01] | 3.62 ± 0.32  [3.22, 4.01] |
|  | Cup | 89.79 ± 0.93  [88.63,90.94] | 81.48 ± 1.53  [79.58,83.38] | 4.25 ± 1.61  [2.25, 6.25] |

**Note: * indicates statistical significance compared with the proposed EEGA-VMUNet (p<0.0056), The Shapiro-Wilk test was used to assess the normality of the data. Paired t-tests were used for normally distributed data, while Wilcoxon signed-rank tests were used otherwise. P-values were adjusted using the Bonferroni correction for multiple comparisons.**

Supplementary Table S6. Performance comparison of different models under 5-fold cross-validation with 95% CI on the REFUGE training set.

| Method | DC(%) (95%CI) | | IoU(%) (95%CI) | HD95(95%CI) |
| --- | --- | --- | --- | --- |
| UNet [23] | Disc | 96.06 ± 0.09*  [95.95,96.18] | 92.43 ± 0.17*  [92.22,92.64] | 6.35 ± 0.25*  [6.04, 6.66] |
|  | Cup | 87.37 ± 0.22*  [87.10,87.64] | 77.57 ± 0.35*  [77.14,78.01] | 9.25 ± 0.20  [9.01, 9.49] |
| AGNet [36] | Disc | 96.21 ± 0.12*  [96.07,96.36] | 92.70 ± 0.22*  [92.43,92.96] | 5.96 ± 0.29*  [5.60, 6.32] |
|  | Cup | 87.69 ± 0.35*  [87.26,88.12] | 78.09 ± 0.55*  [77.40,78.77] | 9.23 ± 0.28*  [8.88, 9.57] |
| MobileNetV3-UNet [39] | Disc | 96.27 ± 0.11*  [96.14,96.40] | 92.81 ± 0.19*  [92.56,93.05] | 6.06 ± 0.29*  [5.70, 6.41] |
|  | Cup | 88.04 ± 0.55*  [87.36,88.73] | 78.65 ± 0.88*  [77.56,79.74] | 8.92 ± 0.29*  [8.55, 9.28] |
| Swin-UMamba [40] | Disc | 96.54 ± 0.08*  [96.45,96.64] | 93.32 ± 0.14*  [93.14,93.50] | 5.40 ± 0.20*  [5.16, 5.65] |
|  | Cup | 88.41 ± 0.64*  [87.62,89.21] | 79.24 ± 1.03*  [77.96,80.52] | 8.81 ± 0.42  [8.28, 9.33] |
| VM-UNet [24] | Disc | 96.26 ± 0.17*  [96.05,96.47] | 92.79 ± 0.31*  [92.41,93.18] | 6.11 ± 0.36*  [5.67, 6.55] |
|  | Cup | 87.76 ± 0.50*  [87.14,88.38] | 78.19 ± 0.79*  [77.21,79.17] | 9.44 ± 0.57  [8.73, 10.14] |
| DA-TransUNet [31] | Disc | 96.47 ± 0.12*  [96.32,96.61] | 93.18 ± 0.22*  [92.91,93.45] | 5.79 ± 0.44*  [5.25, 6.33] |
|  | Cup | 88.31 ± 0.64*  [87.51,89.10] | 79.07 ± 1.03*  [77.79,80.35] | 8.72 ± 0.27*  [8.38, 9.06] |
| MEGANet [28] | Disc | 96.90 ± 0.10*  [96.78,97.02] | 93.98 ± 0.18*  [93.76,94.20] | 4.90 ± 0.27  [4.56, 5.24] |
|  | Cup | 88.70 ± 0.60*  [87.98,89.47] | 79.73 ± 0.97*  [78.53,80.94] | 8.50 ± 0.31  [8.12, 8.88] |
| Res-AttUNet [37] | Disc | 96.28 ± 0.12*  [96.14,96.43] | 92.83 ± 0.22*  [92.56,93.11] | 6.56 ± 1.07  [5.24, 7.89] |
|  | Cup | 88.11 ± 0.57*  [87.40,88.82] | 78.75 ± 0.91*  [77.61,79.88] | 8.88 ± 0.31*  [8.49, 9.26] |
| EE-TransUNet [38] | Disc | 96.60 ± 0.12*  [96.45,96.74] | 93.42 ± 0.23*  [93.14,93.70] | 5.33 ± 0.25*  [5.01, 5.64] |
|  | Cup | 88.40 ± 0.61*  [87.72,89.23] | 79.33 ± 0.98*  [78.12,80.55] | 8.72 ± 0.48  [8.13, 9.31] |
| EEGA-VMUNet | Disc | 97.21 ± 0.10  [97.09,97.32] | 94.56 ± 0.18  [94.34,94.79] | 4.40 ± 0.19  [4.17, 4.63] |
|  | Cup | 88.92 ± 0.61  [88.16,89.67] | 80.06 ± 0.98  [78.84,81.28] | 8.35 ± 0.30  [7.98, 8.73] |

**Note: * indicates statistical significance compared with the proposed EEGA-VMUNet (p<0.0056), The Shapiro-Wilk test was used to assess the normality of the data. Paired t-tests were used for normally distributed data, while Wilcoxon signed-rank tests were used otherwise. P-values were adjusted using the Bonferroni correction for multiple comparisons.**

Supplementary Table S7. P-values of paired tests between EEGA-VMUNet and competing methods across 5 folds on the DRISHTI-GS training set.

| EEGA-VMUNet  VS | DC (p-value) | | | IoU (p-value) | | | | HD95 (p-value) | | |
| --- | --- | --- | --- | --- | --- | --- | --- | --- | --- | --- |
|  | Disc | Cup |  | | Disc | Cup |  | | Disc | Cup |
| UNet | <0.0001 | 0.0001 |  | | <0.0001 | 0.0001 |  | | 0.0023 | 0.0211 |
| AGNet | <0.0001 | 0.0057 |  | | <0.0001 | 0.0051 |  | | 0.0192 | 0.0459 |
| MobileNetV3-UNet | 0.0007 | 0.0008 |  | | 0.0008 | 0.0007 |  | | 0.0022 | 0.0025 |
| Swin-UMamba | 0.0002 | <0.0001 |  | | 0.0002 | <0.0001 |  | | 0.0138 | 0.0429 |
| VM-UNet | <0.0001 | 0.0020 |  | | <0.0001 | 0.0015 |  | | 0.0029 | 0.0126 |
| DA-TransUNet | 0.0072 | 0.0001 |  | | 0.0077 | <0.0001 |  | | 0.0069 | 0.0257 |
| MEGANet | 0.0031 | 0.0463 |  | | 0.0034 | 0.0459 |  | | 0.0044 | 0.0439 |
| Res-AttUNet | 0.0016 | 0.0001 |  | | 0.0018 | <0.0001 |  | | 0.0056 | <0.0001 |
| EE-TransUNet | 0.0095 | 0.0017 |  | | 0.0097 | 0.0018 |  | | 0.0221 | 0.0067 |

**Note: The Shapiro-Wilk test was used to assess the normality of the data. Paired t-tests were used for normally distributed data, while Wilcoxon signed-rank tests were used otherwise. P-values were adjusted using the Bonferroni correction for multiple comparisons (p<0.0056).**

Supplementary Table S8. P-values of paired tests between EEGA-VMUNet and competing methods across 5 folds on the ORIGA training set.

| EEGA-VMUNet  VS | DC (p-value) | | | IoU (p-value) | | | | HD95 (p-value) | | |
| --- | --- | --- | --- | --- | --- | --- | --- | --- | --- | --- |
|  | Disc | Cup |  | | Disc | Cup |  | | Disc | Cup |
| UNet | 0.0003 | 0.0020 |  | | 0.0003 | 0.0022 |  | | 0.0061 | 0.0119 |
| AGNet | 0.0005 | 0.0016 |  | | 0.0006 | 0.0018 |  | | 0.0056 | 0.0125 |
| MobileNetV3-UNet | 0.0003 | 0.0066 |  | | 0.0003 | 0.0075 |  | | 0.0077 | 0.0089 |
| Swin-UMamba | 0.0002 | 0.0026 |  | | 0.0003 | 0.0027 |  | | 0.0207 | 0.0371 |
| VM-UNet | 0.0010 | 0.0033 |  | | 0.0010 | 0.0036 |  | | 0.0070 | 0.0089 |
| DA-TransUNet | <0.0001 | 0.0021 |  | | <0.0001 | 0.0025 |  | | 0.0132 | 0.0055 |
| MEGANet | 0.0030 | 0.0022 |  | | 0.0039 | 0.0023 |  | | 0.0304 | 0.0329 |
| Res-AttUNet | 0.0001 | 0.0042 |  | | 0.0001 | 0.0048 |  | | 0.0116 | 0.0136 |
| EE-TransUNet | 0.0005 | 0.0015 |  | | 0.0006 | 0.0015 |  | | 0.0385 | 0.0323 |

**Note: The Shapiro-Wilk test was used to assess the normality of the data. Paired t-tests were used for normally distributed data, while Wilcoxon signed-rank tests were used otherwise. P-values were adjusted using the Bonferroni correction for multiple comparisons (p<0.0056).**

Supplementary Table S9. P-values of paired tests between EEGA-VMUNet and competing methods across 5 folds on the RIM-ONE-r3 training set.

| EEGA-VMUNet  VS | DC (p-value) | | | IoU (p-value) | | | | HD95 (p-value) | | |
| --- | --- | --- | --- | --- | --- | --- | --- | --- | --- | --- |
|  | Disc | Cup |  | | Disc | Cup |  | | Disc | Cup |
| UNet | 0.0015 | 0.0008 |  | | 0.0014 | 0.0009 |  | | 0.0023 | 0.0225 |
| AGNet | 0.0022 | 0.0010 |  | | 0.0023 | 0.0012 |  | | 0.0066 | 0.0449 |
| MobileNetV3-UNet | 0.0040 | 0.0084 |  | | 0.0040 | 0.0090 |  | | 0.0124 | 0.0165 |
| Swin-UMamba | 0.0055 | 0.0046 |  | | 0.0055 | 0.0045 |  | | 0.0181 | 0.0351 |
| VM-UNet | 0.0016 | 0.0027 |  | | 0.0015 | 0.0030 |  | | 0.0050 | 0.0236 |
| DA-TransUNet | 0.0168 | 0.0027 |  | | 0.0164 | 0.0028 |  | | 0.0444 | 0.0312 |
| MEGANet | 0.0067 | 0.0044 |  | | 0.0059 | 0.0040 |  | | 0.0266 | 0.0440 |
| Res-AttUNet | 0.0076 | 0.0136 |  | | 0.0079 | 0.0141 |  | | 0.0221 | 0.0300 |
| EE-TransUNet | 0.0078 | 0.0139 |  | | 0.0072 | 0.0137 |  | | 0.0033 | 0.0407 |

**Note: The Shapiro-Wilk test was used to assess the normality of the data. Paired t-tests were used for normally distributed data, while Wilcoxon signed-rank tests were used otherwise. P-values were adjusted using the Bonferroni correction for multiple comparisons (p<0.0056).**

Supplementary Table S10. P-values of paired tests between EEGA-VMUNet and competing methods across 5 folds on the REFUGE training set.

| EEGA-VMUNet  VS | DC (p-value) | | | IoU (p-value) | | | | HD95 (p-value) | | |
| --- | --- | --- | --- | --- | --- | --- | --- | --- | --- | --- |
|  | Disc | Cup |  | | Disc | Cup |  | | Disc | Cup |
| UNet | 0.0001 | 0.0019 |  | | 0.0001 | 0.0020 |  | | 0.0002 | 0.0095 |
| AGNet | 0.0001 | 0.0007 |  | | 0.0001 | 0.0008 |  | | 0.0005 | 0.0001 |
| MobileNetV3-UNet | 0.0001 | 0.0001 |  | | 0.0001 | 0.0001 |  | | 0.0002 | <0.0001 |
| Swin-UMamba | 0.0001 | <0.0001 |  | | 0.0001 | <0.0001 |  | | <0.0001 | 0.0271 |
| VM-UNet | 0.0003 | 0.0007 |  | | 0.0003 | 0.0007 |  | | 0.0002 | 0.0060 |
| DA-TransUNet | 0.0001 | <0.0001 |  | | 0.0001 | <0.0001 |  | | 0.0004 | 0.0001 |
| MEGANet | 0.0048 | 0.0001 |  | | 0.0040 | 0.0001 |  | | 0.0142 | 0.0087 |
| Res-AttUNet | <0.0001 | 0.0001 |  | | <0.0001 | 0.0001 |  | | 0.0061 | 0.0042 |
| EE-TransUNet | 0.0006 | 0.0001 |  | | 0.0006 | 0.0001 |  | | 0.0021 | 0.0356 |

**Note: The Shapiro-Wilk test was used to assess the normality of the data. Paired t-tests were used for normally distributed data, while Wilcoxon signed-rank tests were used otherwise. P-values were adjusted using the Bonferroni correction for multiple comparisons (p<0.0056).**

Supplementary Table S11. Pairwise p-values (Bootstrap-based) for EEGA-VMUNet versus competing methods on the DRISHTI-GS test set.

| EEGA-VMUNet  VS | DC (p-value) | | | IoU (p-value) | | | | HD95 (p-value) | | |
| --- | --- | --- | --- | --- | --- | --- | --- | --- | --- | --- |
|  | Disc | Cup |  | | Disc | Cup |  | | Disc | Cup |
| UNet | <0.0001 | <0.0001 |  | | <0.0001 | <0.0001 |  | | <0.0001 | <0.0001 |
| AGNet | <0.0001 | <0.0001 |  | | <0.0001 | <0.0001 |  | | <0.0001 | <0.0001 |
| MobileNetV3-UNet | <0.0001 | 0.0152 |  | | <0.0001 | 0.0213 |  | | <0.0001 | 0.0563 |
| MedSAM | <0.0001 | <0.0001 |  | | <0.0001 | <0.0001 |  | | <0.0001 | 0.1903 |
| Swin-UMamba | <0.0001 | 0.0401 |  | | <0.0001 | 0.0318 |  | | <0.0001 | 0.1337 |
| VM-UNet | <0.0001 | 0.0046 |  | | <0.0001 | 0.0032 |  | | <0.0001 | 0.0066 |
| DA-TransUNet | <0.0001 | 0.0071 |  | | <0.0001 | 0.0152 |  | | <0.0001 | 0.0291 |
| MEGANet | 0.0006 | 0.6260 |  | | 0.0006 | 0.4212 |  | | 0.0003 | 0.6095 |
| Res-AttUNet | <0.0001 | 0.0608 |  | | <0.0001 | 0.0168 |  | | <0.0001 | 0.0990 |
| EE-TransUNet | <0.0001 | 0.4762 |  | | <0.0001 | 0.4202 |  | | <0.0001 | 0.1915 |

**Note: Pairwise p-values were derived from paired bootstrap resampling (1,000 iterations) and adjusted using the Bonferroni correction for multiple comparisons (threshold: p < 0.005).**

Supplementary Table S12. Pairwise p-values (Bootstrap-based) for EEGA-VMUNet versus competing methods on the ORIGA test set.

| EEGA-VMUNet  VS | DC (p-value) | | | IoU (p-value) | | | | HD95 (p-value) | | |
| --- | --- | --- | --- | --- | --- | --- | --- | --- | --- | --- |
|  | Disc | Cup |  | | Disc | Cup |  | | Disc | Cup |
| UNet | <0.0001 | 0.0027 |  | | <0.0001 | 0.0022 |  | | <0.0001 | 0.0038 |
| AGNet | <0.0001 | 0.0031 |  | | <0.0001 | 0.0029 |  | | <0.0001 | 0.0199 |
| MobileNetV3-UNet | <0.0001 | 0.0079 |  | | <0.0001 | 0.0073 |  | | <0.0001 | 0.0148 |
| MedSAM | <0.0001 | <0.0001 |  | | <0.0001 | <0.0001 |  | | <0.0001 | <0.0001 |
| Swin-UMamba | <0.0001 | 0.0206 |  | | <0.0001 | 0.0207 |  | | 0.0022 | 0.0004 |
| VM-UNet | <0.0001 | 0.0204 |  | | <0.0001 | 0.0173 |  | | <0.0001 | 0.1362 |
| DA-TransUNet | <0.0001 | 0.2155 |  | | <0.0001 | 0.1937 |  | | <0.0001 | 0.7784 |
| MEGANet | 0.0079 | 0.4104 |  | | 0.0087 | 0.3958 |  | | <0.0001 | 0.8078 |
| Res-AttUNet | <0.0001 | 0.0219 |  | | <0.0001 | 0.0178 |  | | <0.0001 | 0.0082 |
| EE-TransUNet | <0.0001 | 0.6122 |  | | <0.0001 | 0.5480 |  | | <0.0001 | 0.1668 |

**Note: Pairwise p-values were derived from paired bootstrap resampling (1,000 iterations) and adjusted using the Bonferroni correction for multiple comparisons (threshold: p < 0.005).**

Supplementary Table S13. Pairwise p-values (Bootstrap-based) for EEGA-VMUNet versus competing methods on the RIM-ONE-r3 test set.

| EEGA-VMUNet  VS | DC (p-value) | | | IoU (p-value) | | | | HD95 (p-value) | | |
| --- | --- | --- | --- | --- | --- | --- | --- | --- | --- | --- |
|  | Disc | Cup |  | | Disc | Cup |  | | Disc | Cup |
| UNet | <0.0001 | <0.0001 |  | | <0.0001 | <0.0001 |  | | <0.0001 | <0.0001 |
| AGNet | <0.0001 | <0.0001 |  | | <0.0001 | <0.0001 |  | | <0.0001 | <0.0001 |
| MobileNetV3-UNet | 0.0004 | 0.0017 |  | | 0.0004 | 0.0004 |  | | 0.0033 | 0.0004 |
| MedSAM | <0.0001 | <0.0001 |  | | <0.0001 | <0.0001 |  | | <0.0001 | 0.0249 |
| Swin-UMamba | 0.0147 | 0.0558 |  | | 0.0147 | 0.0273 |  | | 0.0330 | 0.3293 |
| VM-UNet | <0.0001 | <0.0001 |  | | <0.0001 | <0.0001 |  | | <0.0001 | 0.0002 |
| DA-TransUNet | 0.0114 | 0.0117 |  | | 0.0113 | 0.0070 |  | | 0.0060 | 0.0175 |
| MEGANet | 0.0755 | 0.0448 |  | | 0.0764 | 0.0264 |  | | 0.6722 | 0.1317 |
| Res-AttUNet | 0.0012 | <0.0001 |  | | 0.0010 | <0.0001 |  | | 0.0024 | <0.0001 |
| EE-TransUNet | 0.1855 | 0.1622 |  | | 0.1945 | 0.1706 |  | | 0.3159 | 0.5166 |

**Note: Pairwise p-values were derived from paired bootstrap resampling (1,000 iterations) and adjusted using the Bonferroni correction for multiple comparisons (threshold: p < 0.005).**

Supplementary Table S14. Pairwise p-values (Bootstrap-based) for EEGA-VMUNet versus competing methods on the REFUGE test set.

| EEGA-VMUNet  VS | DC (p-value) | | | IoU (p-value) | | | | HD95 (p-value) | | |
| --- | --- | --- | --- | --- | --- | --- | --- | --- | --- | --- |
|  | Disc | Cup |  | | Disc | Cup |  | | Disc | Cup |
| UNet | <0.0001 | 0.0003 |  | | <0.0001 | 0.0002 |  | | <0.0001 | <0.0001 |
| AGNet | <0.0001 | <0.0001 |  | | <0.0001 | <0.0001 |  | | <0.0001 | <0.0001 |
| MobileNetV3-UNet | <0.0001 | <0.0001 |  | | <0.0001 | <0.0001 |  | | <0.0001 | <0.0001 |
| MedSAM | <0.0001 | <0.0001 |  | | <0.0001 | <0.0001 |  | | <0.0001 | <0.0001 |
| Swin-UMamba | <0.0001 | 0.0060 |  | | <0.0001 | 0.0075 |  | | <0.0001 | 0.0015 |
| VM-UNet | <0.0001 | 0.0013 |  | | <0.0001 | 0.0010 |  | | <0.0001 | 0.0012 |
| DA-TransUNet | <0.0001 | 0.0021 |  | | <0.0001 | 0.0025 |  | | <0.0001 | 0.0014 |
| MEGANet | 0.0006 | 0.5564 |  | | 0.0006 | 0.5567 |  | | 0.0003 | 0.1077 |
| Res-AttUNet | <0.0001 | <0.0001 |  | | <0.0001 | <0.0001 |  | | <0.0001 | <0.0001 |
| EE-TransUNet | <0.0001 | 0.0437 |  | | <0.0001 | 0.0439 |  | | <0.0001 | 0.0006 |

**Note: Pairwise p-values were derived from paired bootstrap resampling (1,000 iterations) and adjusted using the Bonferroni correction for multiple comparisons (threshold: p < 0.005).**

Supplementary Table S15. Pairwise p-values (Bootstrap-based) for EEGA-VMUNet versus competing methods on the cross-domain test set.

| EEGA-VMUNet  VS | DC (p-value) | | | IoU (p-value) | | | | HD95 (p-value) | | |
| --- | --- | --- | --- | --- | --- | --- | --- | --- | --- | --- |
|  | Disc | Cup |  | | Disc | Cup |  | | Disc | Cup |
| UNet | 0.0004 | 0.0125 |  | | 0.0004 | 0.0115 |  | | <0.0001 | 0.0084 |
| AGNet | <0.0001 | 0.0179 |  | | <0.0001 | 0.0151 |  | | <0.0001 | 0.0186 |
| MobileNetV3-UNet | 0.0002 | 0.0009 |  | | 0.0002 | 0.0004 |  | | <0.0001 | <0.0001 |
| MedSAM | <0.0001 | 0.0141 |  | | <0.0001 | 0.0231 |  | | 0.0101 | 0.0010 |
| Swin-UMamba | 0.0005 | 0.0257 |  | | 0.0004 | 0.0189 |  | | <0.0001 | 0.0085 |
| VM-UNet | 0.0003 | 0.0270 |  | | 0.0002 | 0.0227 |  | | 0.0001 | 0.0139 |
| DA-TransUNet | 0.0559 | 0.0619 |  | | 0.0501 | 0.0559 |  | | 0.0017 | 0.0370 |
| MEGANet | 0.1682 | 0.1301 |  | | 0.1437 | 0.1206 |  | | 0.0445 | 0.0741 |
| Res-AttUNet | <0.0001 | 0.0453 |  | | <0.0001 | 0.0439 |  | | <0.0001 | 0.0396 |
| EE-TransUNet | 0.0045 | 0.0044 |  | | 0.0036 | 0.0091 |  | | 0.0008 | 0.0037 |

**Note: Pairwise p-values were derived from paired bootstrap resampling (1,000 iterations) and adjusted using the Bonferroni correction for multiple comparisons (threshold: p < 0.005).**

Supplementary Figure S1. Training loss curves on different datasets. (a) DRISHTI-GS, (b) ORIGA, (c) RIM-ONE-r3, and (d) REFUGE.
